# Spatial gene expression at single-cell resolution from histology using deep learning with GHIST

**DOI:** 10.1038/s41592-025-02795-z

**Published:** 2025-09-15

**Authors:** Xiaohang Fu, Yue Cao, Beilei Bian, Chuhan Wang, Dinny Graham, Nirmala Pathmanathan, Ellis Patrick, Jinman Kim, Jean Yee Hwa Yang

**Affiliations:** 1 School of Mathematics and Statistics, The University of Sydney, Sydney, New South Wales Australia; 2 School of Computer Science, The University of Sydney, Sydney, New South Wales Australia; 3 Sydney Precision Data Science Centre, University of Sydney, Sydney, New South Wales Australia; 4 Charles Perkins Centre, The University of Sydney, Sydney, New South Wales Australia; 5 Laboratory of Data Discovery for Health Limited (D24H), Pak Shek Kok, Hong Kong SAR China; 6 Centre for Cancer Research, The Westmead Institute for Medical Research, Sydney, New South Wales Australia; 7 Westmead Breast Cancer Institute, Westmead Hospital, Western Sydney Local Health District, Sydney, New South Wales Australia; 8 Faculty of Medicine and Health, University of Sydney, Sydney, New South Wales Australia; 9 Douglass Hanly Moir Pathology, Sydney, New South Wales Australia

**Keywords:** Machine learning, Image processing, Computational models, Gene expression, Functional genomics

## Abstract

The increased use of spatially resolved transcriptomics provides new biological insights into disease mechanisms. However, the high cost and complexity of these methods are barriers to broader application. Consequently, methods have been created to predict spot-based gene expression from routinely collected histology images. Recent benchmarking showed that current methodologies have limited accuracy and spatial resolution, constraining translational capacity. Here, we introduce GHIST, a deep learning-based framework that predicts spatial gene expression at single-cell resolution by leveraging subcellular spatial transcriptomics and synergistic relationships between multiple layers of biological information. We validated GHIST using public datasets and The Cancer Genome Atlas data, demonstrating its flexibility across different spatial resolutions and superior performance. Our results underscore the utility of in silico generation of single-cell spatial gene expression measurements and the capacity to enrich existing datasets with a spatially resolved omics modality, paving the way for scalable multi-omics analysis and biomarker identification.

## Abstract (web summary)

GHIST is a deep learning-based method that can predict spatial gene expression at high resolution using histology data.

## Main

Spatially resolved transcriptomics (SRT) profiling technologies provide spatially resolved mapping of gene expression and hold the potential to revolutionize our understanding of multicellular biological systems. SRT can enable new insights to improve the prediction of key clinical outcomes of complex diseases, including cancers^1–4^. The recent subcellular in situ imaging-based SRT, herein referred to as subcellular spatial transcriptomics (SST), platforms (for example, 10x Xenium^5^, NanoString CosMx^6^ and Vizgen MERSCOPE) can map spatial gene expression (SGE) at subcellular resolution. Spot-based SRT platforms (for example, 10x Visium) typically capture the gene expression of multiple cells per spot, generating spot-based SGE. In contrast, the newer SST data are assayed at a resolution smaller than the typical cell and are typically passed through a cell segmentation workflow to provide ‘single-cell SGE’. This finer spatial resolution of gene expression unlocks the capacity to explore a variety of hypotheses that were previously inaccessible and provide deeper biological insights.

Despite the breakthrough capabilities in SRT, the high costs associated with these technologies hinder broader application. On the other hand, histopathology images, such as hematoxylin-and-eosin (H&E)-stained images, are routinely collected and widely available. H&E-stained images contain rich visual details on cellular and tissue structure that reflect the integration of the underlying biological information in the tissue^7^. The capacity to predict SGE from histopathology alone would unlock insights into variation in gene expression and cell-type-specific expression that were previously unattainable from the large library of H&E images available. This can provide new opportunities for exploring cellular heterogeneity and the molecular patterning underpinnings of tissue organization.

Deep learning-based methods have been developed to facilitate the prediction of spot-level spatial profiles of gene expression values from H&E-stained images^8–14^. They include ST-Net, Hist2ST, HisToGene, DeepPT and BLEEP. These methods learn visual and spatial associations between matched H&E images and spot-level SGE data. Existing methods typically examine H&E image patches with a convolutional neural network^8,10,12^ or Transformer^9,11^ backbone. For example, ST-Net leveraged a convolutional neural network-based backbone to extract an embedding of image patches that corresponded to spots, then applied a fully connected layer to predict gene expression of each spot. Some methods further examined the relationships between adjacent spots on a more global level, for example with graph neural networks. For example, Hist2ST combined a Convmixer, Transformer and a graph neural network to extract patch-based features, and considered the spatial relationships among the spots to enhance spatial dependencies from the entire image. Furthermore, an alternative strategy is to apply super-resolution to spot-based data^14^. Super-resolved pixels, however, potentially contain a mixture of cells with background areas, and are thus not at true single-cell resolution. Our previous benchmarking study^15^ on the existing methods revealed a weak translational potential, which is likely due to the limited information in the predictions. In these results, gene expression in each spot describes a mixture of cells and a mixture of cell types, which contain limited power as spot-based SGE is not at single-cell resolution. None of these methods have formulated the spatial associations to leverage subcellular resolution SGE data from newer platforms such as 10x Xenium. Methods have been developed to leverage histology and SGE data to predict high-resolution SGE; however, they require input spot-based SGE data during prediction, thereby limiting wider applicability^16–18^.

The increase in resolution from spot-level to single-cell level poses a substantial challenge, as this presents an increased number of expression profiles (from hundreds to thousands of spots, to over tens of thousands of cells) and necessitates the ability to discern finer-grained variations in H&E images that correspond to subtle variations in gene expression. These fine-grained details in H&E images are typically noisy and may provide limited information for discerning patterns that are even broader than gene expression, such as cell types. Different cell types that express distinct gene markers and typically exhibit similar morphological features in histology may be difficult to tell apart with morphological information alone, for example, some types of lymphocytes including B cells and T cells. Additionally, gene expression within a target cell is known to be influenced by neighboring cell types, for example, various fibroblast subtypes that occur in the presence of macrophages or malignant cells. Overall, there are complex relationships between the histological appearance of cells, gene expression within cells, cell type and composition of neighboring cell types. A deep learning model can be designed to leverage these relationships to achieve better gene expression predictions. To our knowledge, there is not yet a method that is specifically designed for single-cell SGE prediction from histology images, and the relationships between these multiple layers of biological information into such a method remain to be leveraged.

**Fig. 1:**
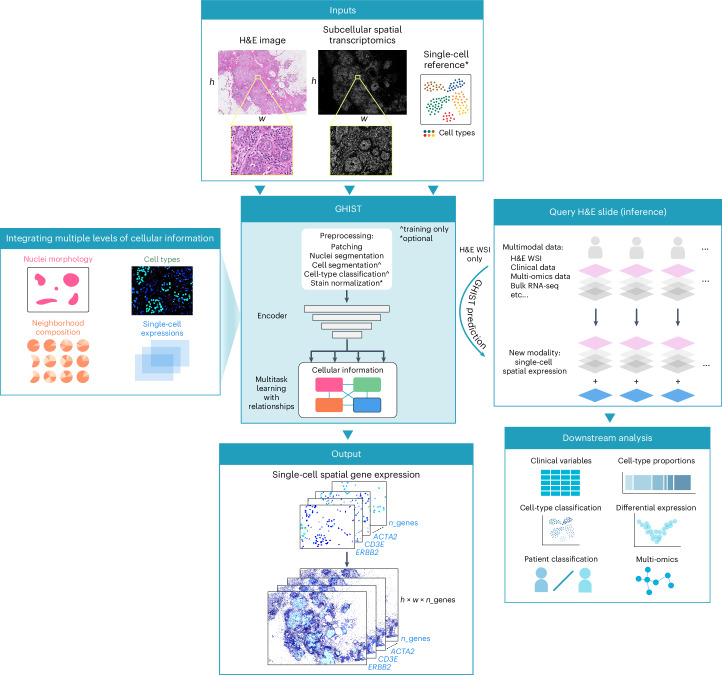
GHIST framework. GHIST maps H&E images to spatially resolved single-cell gene expression through leveraging paired SST data during training. The multitask deep learning-based model integrates relationships between multiple levels of information related to gene expression within cells. Once trained, GHIST enables the in silico estimation of spatially resolved single-cell gene expression from existing H&E data alone, which can be used to enrich further downstream analysis. The cell segmentation and cell-type classification preprocessing steps are performed during training only. During inference, stain normalization is applied on query H&E slides. ^ denotes training only and * denotes optional input or step. WSI, whole-slide image.

Here, we introduce a new framework to predict spatially resolved single-cell Gene expression from HISTology, GHIST (pronounced ‘dʒɪst’; Fig. 1). GHIST addresses the challenges of predicting single-cell SGE from histology images through a number of key innovations. Most importantly, GHIST leverages multiple prediction components that use the relationships between multiple layers of information (including cell type, neighborhood composition, nuclei morphology and single-cell expression). GHIST synergistically captures interdependencies between information types by its multitask architecture and multiple loss functions, which overall improves SGE prediction. Furthermore, GHIST is designed to handle different resolutions in addition to single-cell-level SGE, such as spot-based SGE, for which we demonstrate the superior performance of GHIST compared to the state-of-the-art approaches. Along with the development of our method, we demonstrate the power of GHIST in providing an innovative in silico strategy to add a layer of rich spatial information to existing large-scale databases, without costly practical experiments. This can facilitate the generation of new hypotheses for spatial experiments, the discovery of cell-type-specific and spatial associations between biomarkers and relationships that provide new insights into disease mechanisms.

## Results

### GHIST: single-cell Gene expression from HISTology

GHIST is a multitask deep learning-based learning method that enables spatially resolved SGE prediction at single-cell-level resolution by mapping H&E images to the expression of hundreds of genes within individual cells in the image, and providing spatial localization of cells (Fig. 1). In other words, GHIST maps H&E images to a collection of SGE images (where the number of channels equals the number of genes, and pixel intensities equal the total expression of individual genes within cells). GHIST can achieve this mapping by learning from samples comprising an H&E image and its corresponding SST data. Once trained, SST data are not used during inference of SGE. In other words, the trained model is applied to unseen H&E data only and no additional spatial omics-level information is required. During training, single-cell RNA sequencing (RNA-seq) data are leveraged to improve the predicted expression profiles by providing known cell-type information and may be extracted from public repositories such as the Human Cell Atlas or CZI CELLxGENE. There is no requirement for the single-cell data to be matched to the input H&E images.

GHIST considers the interdependencies between four levels of biological information, including (i) cell type, (ii) neighborhood composition, (iii) cell nucleus morphology and (iv) single-cell RNA expression. GHIST leverages its multitask deep learning architecture with four prediction heads (corresponding to the information types) to jointly learn these types of information for each cell. Features and predictions provided by each prediction head are used between different prediction heads, as an input or through training losses that help the model to learn the interdependencies. The design of the prediction heads and loss functions is informed by biological knowledge to capture interdependencies between information types. For example, the composition of the local neighborhood is used to inform gene expression prediction, and gene expression within cells is used to predict cell type to ensure the expression is biologically meaningful (Methods). This design allows GHIST to elucidate the variations in gene expression within single cells from H&E images, and enables better predictions of gene expression for cell types that are difficult to distinguish in histology (Methods and Supplementary Figs. 1 and 2).

### GHIST effectively captures single-cell spatial expression

**Fig. 2:**
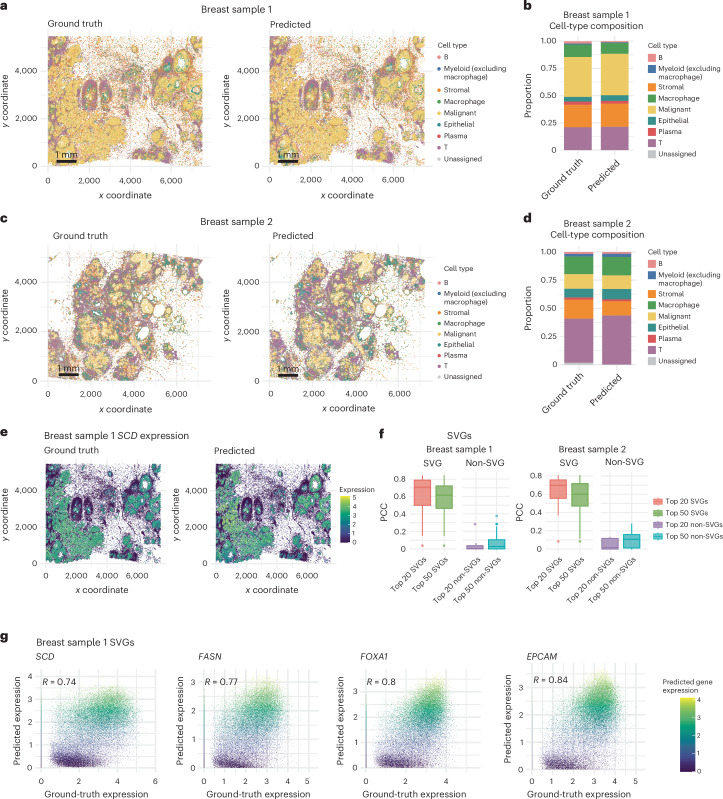
GHIST predictions on two breast cancer H&E images. **a**,**b**, Comparison between cell types (**a**) and cell-type compositions (**b**) obtained from BreastCancer1 paired SST (Xenium) data and predicted cell types from expression predicted from H&E. **c**,**d**, Comparison between cell types (**c**) and cell-type compositions (**d**) obtained from BreastCancer2 paired SST (Xenium) data and predicted cell types from expression predicted from H&E. Scale bar, 1 mm. **e**, Predicted gene expression in individual cells from GHIST and SST data (for the gene *SCD*) on BreastCancer1. **f**, Box plot of the computed PCC between predicted and measured (ground truth) expression for the top 20 and 50 predicted SVGs and non-SVGs in both BreastCancer1 and BreastCancer2. Each box plot ranges from the first to third quartile with the median as the horizontal line. The lower whisker extends to 1.5 times the interquartile range below the first quartile, while the upper whisker extends to 1.5 times the interquartile range above the third quartile. The sample size corresponds to the number of genes included (either 20 or 50). **g**, Scatterplots showing the predicted SGE expression of *SCD*, *FASN*, *FOXA1* and *EPCAM* compared to measured SST expression. Source data

We used a range of evaluation metrics including cell-type proportion, spatially variable genes (SVGs) and correlation to evaluate the reliability of the gene expression predicted by GHIST. Here we define the ‘ground-truth’ single-cell expression values as those obtained through carrying out cell segmentation on SST data, while the cell types were obtained by applying a standard supervised cell annotation tool, scClassify, on the expression (Methods). Figure 2a–d and Supplementary Fig. 3 compare the cell types predicted based on the predicted expression from GHIST to the ground-truth cell types for two breast samples. The cell-type distributions on the slides (Fig. 2a,c), including overall cell-type composition (Fig. 2b,d), were strikingly similar between the ground truth and the predictions, showing that the predicted gene expression by GHIST successfully maintained cell-type information of the samples. For this multi-class (eight cell-type classes) prediction problem, the cell-type accuracy was 0.75 for BreastCancer1 and 0.66 for BreastCancer2.

Next, we examined if the predicted expression values are accurate by examining the correlation between predicted and ground-truth expression among SVGs or highly variable genes (HVGs). Using stearoyl-CoA desaturase 1 (*SCD*) as an example, we observe a strong agreement between the ground-truth and predicted gene expression across the slide (Fig. 2e,f). We calculated the correlation between the predicted and measured (ground truth) intensities for the SVGs and the non-SVGs. We found a high correlation between the predicted and measured expression of SVGs, representing biologically meaningful genes, with the top 20 and top 50 SVGs having median correlations of 0.7 and 0.6, respectively (Fig. 2f). In contrast, as we do not anticipate correlation between two sets of white noise data, genes with low expression should not have correlation between predicted and measured intensities. Consequently, among the non-SVGs, which included many low-expressed genes, we observed a very low correlation of 0 to 0.1, as expected. Some highly correlated SVGs included *SCD* (*r* = 0.74), *FASN* (*r* = 0.77), *FOXA1* (*r* = 0.8) and *EPCAM* (*r* = 0.84; Fig. 2g), which are meaningful genes with known associations with breast cancer^19–22^. Similar results were observed when we focused on HVGs (Supplementary Fig. 4a,b). Finally, GHIST was applied to two additional datasets—LungAdenocarcinoma and Melanoma—for which strong agreement between predicted and ground-truth cell-type proportions was observed (Supplementary Figs. 5 and 6; melanoma *r* = 0.92 and lung adenocarcinoma *r* = 0.97), demonstrating that GHIST is applicable to other cancer tissues.

### GHIST outperforms existing methods on spot-based data

**Fig. 3:**
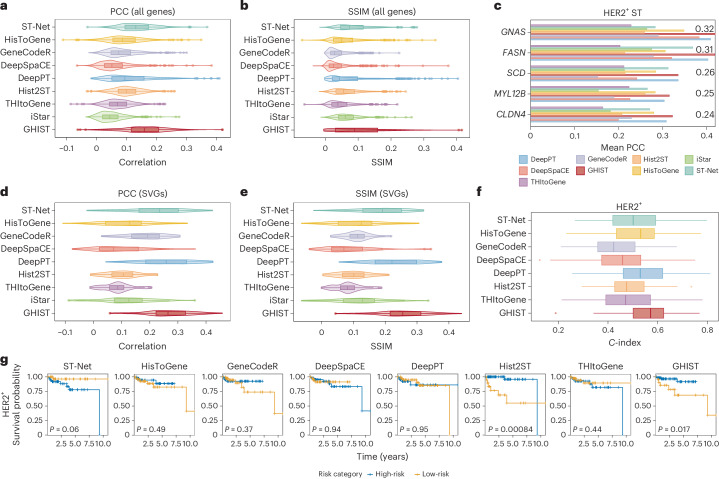
Comparison of spot-based gene prediction and survival analysis performance among state-of-the-art methods and GHIST using the HER2ST dataset. **a**,**b**, Violin and box plots of the average PCC (**a**) and SSIM (**b**) between ground-truth gene expression and predicted gene expression. Metrics measured from the test fold of a fourfold cross-validation, averaged over each gene (*n* = 785) across the dataset. **c**, Top five correlated genes. **d**,**e**, PCC (**d**) and SSIM (**e**) violin and box plots for each method for selected SVGs (*n* = 20 per image sample). **f**, *C*-indices of multivariate cox regression models predicting survival of HER2^+^ subtype from TCGA-BRCA patients (*n* = 92), using RNA-seq bulk, RNA-seq bulk using only genes present in HER2ST dataset, and the predicted pseudobulk from each method. *C*-indices were calculated from the test sets of a threefold cross-validation with 100 repeats. **g**, Cross-validated Kaplan–Meier curves for patients split into high-risk and low-risk groups by the median risk prediction of the multivariate cox regression models for each method and HER2^+^ breast cancer subtypes. The *P* value represents the result of the two-sided log-rank test for assessing the statistical significance of differences in survival between the groups. In **a**, **b** and **d**–**f**, each box plot ranges from the first to third quartile with the median as the horizontal line. The lower whisker extends to 1.5 times the interquartile range below the first quartile, while the upper whisker extends to 1.5 times the interquartile range above the third quartile. Source data

GHIST can be easily adapted to predict spot-based SGE. Here, we used an existing benchmarking framework proposed for spot-based methods^15^ to establish the extensibility of our method. Using the human HER2^+^ breast tumor (HER2ST) dataset^23^, we first evaluated the spot-based SGE predictions with metrics commonly used in the literature such as the Pearson correlation coefficient (PCC) and the structural similarity index (SSIM) across all genes for each method. Figure 3a,b shows that our method achieved the highest PCC of 0.16 (compared to 0.14 for ST-Net and 0.11 for DeepPT), and SSIM of 0.10 (compared to 0.08 for ST-Net and 0.07 for DeepPT). Next, we note that the performance of our method on the top five biologically meaningful genes (Fig. 3c) had the highest correlation compared to other methods (*GNAS* (*r* = 0.42), *FASN* (*r* = 0.42), *SCD* (*r* = 0.34), *MYL12B* (*r* = 0.32) and *CLDN4* (*r* = 0.32)), indicating one should examine correlation among genes with meaningful biological signal. Thus, with the limitation of the PCC values in mind, we examined more meaningful gene characteristic metrics, as discussed in our recent benchmarking work^15^, based on the top 10% of HVGs and top 20 SVGs. Figure 3d,e and Supplementary Fig. 7 show that our method provided the highest PCC in HVGs (0.20) and SVGs (0.27) and SSIM in HVGs (0.17) and SVGs (0.26). Furthermore, the root mean squared error of GHIST was 0.20 for all genes and 0.22 for SVGs (Supplementary Fig. 8). Together, these results demonstrate that GHIST-predicted SGEs that were more aligned to the ground-truth SGE than existing methods and that it improves over existing spot-based methods.

Furthermore, we compared GHIST to iStar^14^. Since iStar does not capture gene expression at single-cell resolution, unlike GHIST, it is not meaningful to directly compare GHIST to iStar, so we compare the two methods at reduced resolutions on two different datasets. GHIST outperformed iStar on the spot-based HER2ST data (Fig. 3a–e). We also performed pseudospot-based comparison using BreastCancer2. GHIST demonstrated superior performance over iStar for the top 20 SVGs, showing stronger correlations with ground-truth expression (Supplementary Fig. 9). Additionally, for genes lacking signal, GHIST effectively illustrated a much lower, or even zero, correlation, avoiding any unintended ‘correlation’ artifacts.

Moreover, we tested the translational potential of predicted SGE for downstream applications using selected individuals from the The Cancer Genome Atlas Breast Invasive Carcinoma (TCGA-BRCA) dataset. Here we converted the predicted SGE to pseudobulk gene expression for each patient, and built multivariate Cox regression models from the pseudobulk gene expression to predict survival using a threefold cross-validation with 100 repeats (Fig. 3f). The corresponding RNA-seq and a subset (RNA-Seq-STgene), comprising only genes present in the HER2ST dataset, were used as baselines for survival analysis. GHIST achieved the highest average *C*-index of 0.57, compared to 0.55 for RNA-Seq-STgene. Next, we binarized the risk model predictions of each patient and split them into high-risk and low-risk groups based on the median risk prediction, then constructed Kaplan–Meier survival curves (Fig. 3g). GHIST showed translational potential by demonstrating a significant difference in survival profiles between individuals from different risk categories (*P* = 0.017).

### GHIST is scalable and translatable on external datasets

**Fig. 4:**
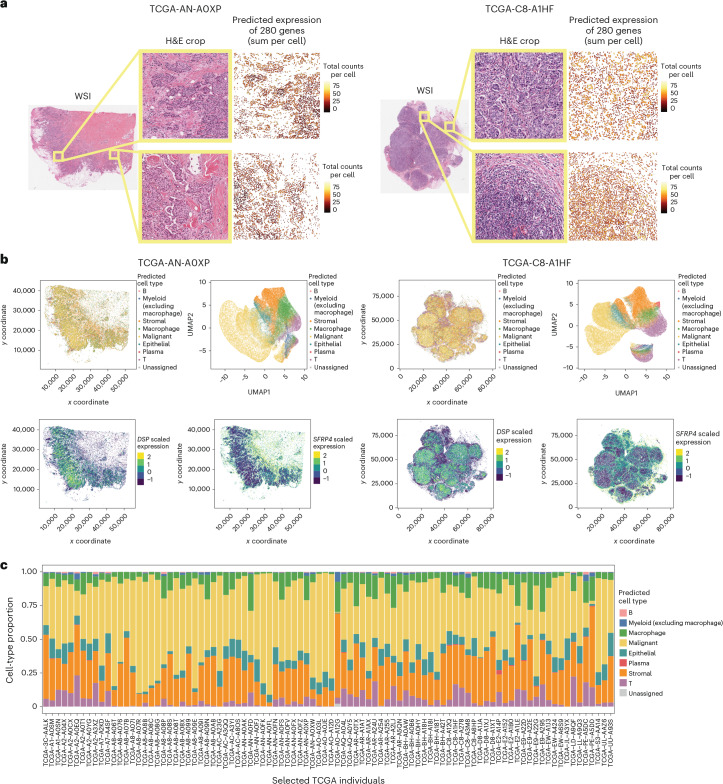
Application of GHIST to TCGA breast cancer H&E images. **a**, Illustration of the capacity of GHIST to predict gene expression for each cell in an H&E WSI (example from two TCGA-BRCA samples, TCGA-AN-A0XP and TCGA-C8-A1HF). **b**, Visualization of the predicted cell type on the two selected TCGA samples. A UMAP visualization is used to project the cells on a two-dimensional plot. A malignant cell-type marker *EPCAM* and a stromal cell-type marker *SFRP4* are used to visualize the GHIST-predicted location of the two cell types. **c**, Predicted cell-type proportions for the selected TCGA HER2^+^ individuals. Source data

To examine the GHIST model in practice, we applied the model in its default single-cell expression setting directly to two external datasets: (1) a public TCGA-BRCA HER2-positive cohort consisting of 92 individuals and a TCGA-BRCA luminal cohort consisting of 461 individuals (defined by patient metadata; Methods), and (2) an in-house breast cancer tissue data consisting of 44 individuals. The TCGA dataset is a public multi-omics collection, containing histological images, bulk RNA-seq data, whole-genome sequencing data and other information such as proteomics. We used the histological image as the input to obtain predicted expression of 280 genes in individual cells. Figure 4a illustrates the predicted expression for H&E patches from two individual samples. We show the uniform manifold approximation and projection (UMAP) of the cell-type compositions for each individual in Fig. 4b. Next, we examined the location of the expression for two marker genes—*EPCAM*, which is a marker of malignant cells, and *SFRP4*, which is more highly expressed in the adjacent tumor microenvironment (Fig. 4b and Supplementary Fig. 10). These observations are consistent with the general assumption that the tumor exists as a confined structure, while other cell types such as fibroblasts and macrophages form the adjacent tumor microenvironment. Distribution of the marker gene expression for each cell type is illustrated in Supplementary Fig. 11. Figure 4c shows the overall cell-type proportion for all 92 HER2^+^ individuals, consisting mostly of malignant cells, followed by stromal cells as expected. Two individuals had minimal predicted malignant cells, and we found their H&E slides to have poor stain quality as assessed by a pathologist (Supplementary Fig. 12, Supplementary Table 1 and Methods). The in-house breast cancer tissue data contain tumor cores collected from both in situ and the adjacent invasive region from the same individual. We characterized the tumor–immune microenvironment using mixing score, and found overall invasive regions tended to have a higher degree of immune infiltration compared to in situ regions (Supplementary Fig. 13). This finding is consistent with the literature^24,25^ and demonstrates the relevance of the GHIST-predicted data.

### An in silico modality for multi-view, multi-omics analysis

**Fig. 5:**
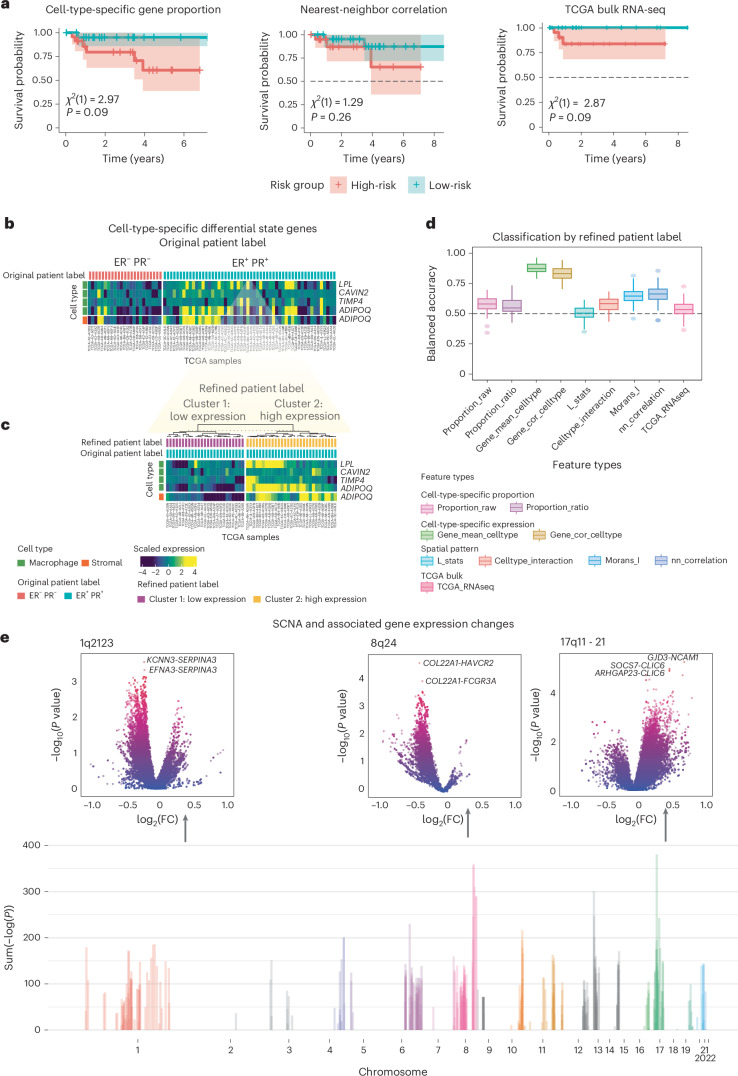
Potential of GHIST to create an in silico modality for multi-view analysis. **a**, Cross-validated Kaplan–Meier curves for TCGA HER2 individuals split into high-risk and low-risk groups by the median risk predictions from cell-type-specific gene proportion, nearest-neighbor correlation and RNA-seq data downloaded from TCGA. Shaded regions represent 95% confidence intervals. A two-sided log-rank test was used to calculate the *χ*² (chi-squared) test statistic and *P* values of the survival difference between the two groups (*n* = 92). **b**, Cell-type-specific differential state genes in macrophage and stromal cells between ER^+^/PR^+^ and ER^−^/PR^−^ patients. **c**, The ER^+^/PR^+^ group exhibited heterogeneity in expression of *LPL*, *CAVIN2*, *TIMP4* and *ADIPOQ*. The clustering method grouped them into two clusters. **d**, We used the various feature types, that is, cell-type proportion, cell-type-specific expression and spatial features extracted from the predicted gene expression, to build a patient outcome prediction model with the two clusters of ER^+^/PR^+^ status refined in **b** as the patient outcome. Higher accuracy indicates better ability to distinguish the two clusters of ER^+^/PR^+^ individuals (*n* = 54). Each box plot ranges from the first to third quartile with the median as the horizontal line. The lower whisker extends to 1.5 times the interquartile range below the first quartile, while the upper whisker extends to 1.5 times the interquartile range above the third quartile. **e**, Differential SGE affected by CNA was calculated using two-sided *t*-test. Volcano plots display three selected hotspots (1, 8, 17q) that affect a number of spatial expression patterns of genes (top). The sum of −log_10_(*P*) of each genomic region of the associations between CNAs and SGE (bottom). The *P* value was reported without multiple-comparison adjustment. FC, fold change. Source data

Using GHIST, we generated spatially resolved single-cell expression of the TCGA individuals, thereby contributing a new modality to the TCGA multi-omics collection. Next, we demonstrate the analytical potential of GHIST through multiple downstream analyses, such as survival analysis and differential state expression, using predicted single-cell data. Using the same selected subset of TCGA HER2^+^ individuals, we examined if the predicted gene expression is associated with survival outcomes. Firstly, we investigated the different types of features that can be extracted from the SGE predicted by GHIST. We revealed that the cell-type information and the SGE pattern enabled by GHIST was able to differentiate high-risk and low-risk individuals (Fig. 5a) in the TCGA HER2^+^ cohort. The cell-type-specific gene proportion (chi-square = 2.97, *P* value = 0.09) separated the risk groups with similar power as the TCGA bulk RNA-seq (chi-square = 2.87, *P* value = 0.09). Notably, in the luminal cohort, with a larger sample size, the nearest-neighbor correlation (chi-square = 7.22, *P* value = 0.01) and Moran’s *I* (chi-square = 6.04, *P* value = 0.01), which both compute the spatial patterning of genes, achieved greater statistical significance than the TCGA bulk RNA-seq (chi-square = 1.9, *P* value = 0.17; Supplementary Fig. 14).

Next, we examined disease subtype detection by comparing estrogen receptor-positive (ER^+^)/progesterone receptor-positive (PR^+^) and estrogen receptor-negative (ER^−^)/progesterone receptor-negative (PR^−^) individuals, which are known to have distinct survival outcomes^26^ (Fig. 5b). In the TCGA HER2^+^ cohort, cell-type-specific analysis uncovered differential state genes specific to macrophage and stromal cells, such as *LPL*, *CAVIN2*, *TIMP4* and *ADIPOQ*. We further observed that these genes exhibited heterogeneity within the ER^+^/PR^+^ group, where one subgroup had lower expression similar to the ER^−^/PR^−^ group, while the other subgroup had higher expression. We show that the high and low expression pattern is not a technical artifact through volcano plots, where there are a similar number of upregulated and downregulated genes between cluster 1 and cluster 2 for all cell types (Supplementary Fig. 15). Figure 5c shows the results of unsupervised clustering on the ER^+^/PR^+^ individuals, demonstrating that cell-type-specific information enabled us to uncover patient heterogeneity within the ER^+^/PR^+^ population. In cluster 2, the higher expression of *LPL*, *CAVIN2* and *TIMP4* within macrophage cells, and *ADIPOQ* in stromal cells, was associated with better survival (Supplementary Fig. 16a), consistent with the literature^27^. Conversely, lower expression was associated with poorer survival. This finding is consistent with the functions of these genes, where lower expression implies impaired metabolic and immune functions.

To further investigate whether there exists potential differential patterns between these two clusters, we used a classification approach to assess the discriminatory potential of spatial patterns compared to cell-type-specific expression patterns. We used scFeatures^28^ to generate a series of feature types and revealed that spatial metrics for differential spatial patterns, such as Moran’s *I* and nearest-neighbor correlation (Moran’s *I* accuracy = 0.64 and nearest-neighbor correlation accuracy = 0.66), also exhibited discrimination capacity (Fig. 5d). As expected, cell-type-specific gene expression (accuracy = 0.87) has a strong discrimination capacity as the clusters were initially defined by this measure. The spatial pattern-based feature type, nearest-neighbor correlation, captures the composition and distribution of cell types in various local regions. Features that demonstrate differential patterning (as defined by nearest-neighbor correlation) include *TNFRSF17*, *CD19*, *KRT23*, *KRT7* and *TRAF4* (Supplementary Fig. 16b), which all have a higher expression correlation with neighboring cells in cluster 2, corresponding to better survival in individuals. These findings highlight the power of GHIST in uncovering additional spatial insights beyond the bulk gene expression data available in TCGA.

### GHIST reveals genomic regions that affect spatial pattern

Somatic copy number alteration (SCNA) has been revealed as a prognostic biomarker for breast cancer, and can often result in gene expression changes^29,30^. We extended the concept of differential expression analysis to define differential patterning genes as genes showing a difference in nearest-neighbor correlation between samples of interest, where nearest-neighbor correlation reflects the degree of spatial clustering or dispersion of different cell types. Here, we investigated the effect of gene gain/loss on spatial patterning of gene expression. First, we selected somatic copy number genes (with gain frequency ≥ 5 and loss frequency ≥ 5 in 91 HER2^+^ individuals; one individual did not have copy number alteration (CNA) data) from the whole genome, which resulted in 2,177 genes in total. Next, for each of the SCNA genes, we performed a differential patterning analysis to identify differential patterning genes between samples showing gene gain versus samples showing gene loss for each SCNA based on the predicted expression of the 280 genes. We identified *trans*-acting hotspots associated with a large number of differential patterning genes in HER2 (Fig. 5e and Supplementary Table 2). These differential patterning hotspots include loci on chromosomes 1, 8 and 17q. Our results recapitulated some well-known genomic regions that have been previously reported to be associated with cancer risk and gene expression. For example, the differential patterning hotspots on chromosome 8 are around the known ‘8q24 gene desert’ that has been associated with prostate, colon and breast cancer^31–34^. Other differential patterning hotspots on chromosome 17 were located in 17q11-21, which is the amplified region containing HER2. We observe that gain/loss *TOP2A* is a strong signal for differential patterning genes (Fig. 5e and Supplementary Table 3). *TOP2A* is a cancer chemotherapy target^35^, and its copy number variation has been revealed as a predictive biomarker of chemotherapy of breast cancer^36,37^. We also performed the same analysis on individuals in the luminal cohort and found that hotspots on chromosome 17 were the strongest signal (Supplementary Fig. 17). CNAs on chromosome 17 were also found to be the most frequent alteration for individuals in the luminal cohort^38^. Taken together, our results suggest that the predicted SGE pattern is a powerful new modality that enables previously unexplored questions relating CNAs and differential patterning.

## Discussion

Here we presented GHIST, a new approach for predicting single-cell SGE from histology images. GHIST leverages multitask deep learning strategies to map H&E images (three channels, RGB) to a collection of cell-level SRT images, by leveraging joint learning of cell nuclei morphology, cell-type information, neighborhood information and gene expression. GHIST is a new method for prediction at single-cell resolution. We demonstrated that GHIST can also be applied to spot-based resolution data and is able to outperform state-of-the-art spot-based approaches. We then showed that GHIST can generate spatially resolved gene expression at single-cell level for breast cancer images in TCGA in silico, thereby contributing a new modality to enrich downstream analysis.

The TCGA-BRCA samples have currently been assayed by ten different experimental strategies, including genomics, transcriptomics and proteomics. Our spatially resolved gene expression measurements complement these existing modalities, providing a spatial omics modality across all of the samples in the collections. Further to this, H&E is a ubiquitous, routinely used histological technique applied in standard clinical practice to provide prognostic information that informs clinical management of nearly all cancers. As such, H&E is widely available with detailed clinical information in our health systems. GHIST can enrich these large collections of existing H&E data by enabling large-scale generation of spatially resolved gene expression profiles, facilitating comprehensive analysis and discoveries. As high-quality training datasets continue to be generated, there is a clear opportunity to increase the availability of this additional spatial modality for other cancer types in the TCGA, and other databases and sample collections. This will enable researchers to investigate SGE patterns to formulate hypotheses about disease mechanisms and cell interactions or, alternatively, allow researchers to pre-examine potential results in silico before conducting costly and time-consuming experiments.

Many current H&E approaches are limited in their ability to identify a diverse range of cell types^39–41^. For instance, the dataset created by the Breast Cancer Segmentation Grand Challenge^42^ focuses on discriminating four key cell types: tumor, stroma, inflammatory and necrosis. In contrast, we can predict double the number of cell types using our approach. This expanded capability provides a more detailed and nuanced understanding of the tissue microenvironment. It is important to note that the further expansion of identifiable cell types is tightly linked to the availability of data and the design of gene panels. Compared to scRNA-seq data, where thousands of genes are measured and many cell types are marked by the expression of dozens of genes, spatial transcriptomics gene panels with 300–500 nonspecific genes may limit our capacity to discriminate cell types if marker genes are not present. For example, in our application to breast cancer, identifying myoepithelial cells, the absence of which distinguish in situ from invasive ductal carcinoma, is challenging because commonly stained markers such as *P63* are not available in the panel. This highlights the importance of customized gene panel design, a service offered by most vendors, and the power of newly emerging algorithms in this area (for example, geneBasis^43^ and gpsFISH^44^). We believe that incorporating many of these considerations going forward by our community will greatly enhance the precision and utility of SGE predictions, allowing for more comprehensive and accurate tissue analysis.

The power of SGE prediction also lies in its potential to gain deeper insights into disease mechanisms through additional spatial information. This allows us to determine not only whether a particular gene is expressed but also where it is expressed and its relative patterning. Such capability enables us to explore whether specific gene expression patterns can further delineate breast cancer subtypes. Our results show that the spatial features including the L statistic (representing the spatial association of cell types), Moran’s *I* (representing the spatial patterning of gene expression) and nearest-neighbor correlation (representing the correlation of genes with neighboring cells), achieved high discriminating power in both survival and classification analysis. Such exploration would not be possible with the existing bulk RNA-seq data in TCGA. These findings underscore the power of GHIST in enabling researchers to examine histology images with a spatial resolution that was previously unattainable.

A limitation identified during the application of GHIST to TCGA data is the variability in H&E slide quality, such as inconsistent staining, which can reduce model performance. Thus, there is a need to account for the quality of H&E slides before the application of our model, as interpretation of suboptimal slides should be avoided, per current histopathology practice. It is known that the quality of H&E staining is dependent on artifacts that are attributed to multiple factors from formalin fixation to tissue processing, including thick and thin sectioning, processing temperatures and times, and batch effects from different tissue processors^7^. To increase utility, there is potential to optimize various aspects of H&E protocols with the aim of increasing accuracy associated with SGE prediction. This includes both wet lab protocols, such as standardizing staining techniques, optimal fixation and implementing rigorous quality-control measures and computational approaches implementing advanced image preprocessing such as stain normalization and leveraging foundation models trained on a large number of H&E images. Integrating these optimized protocols will lead to more reliable identification of spatial patterns in gene expression and deeper insights into disease mechanisms.

Although GHIST demonstrated strong discriminative performance when trained on a limited dataset, the restricted amount of data remains a limitation of the current study. During training, the model used thousands of high-resolution patches from a slide containing about 80,000 cells, which allowed the model to capture intricate details and subtle variations, thus ensuring strong practical performance even when trained on very limited samples. While the number of slides was limited, we were effectively learning from a larger number of matched cells from H&E images and gene expression measurements. This contributes to the model’s ability to generalize across H&E images from different cohorts, leading to robust and reliable predictions in real-world applications. We anticipate an increase in model robustness with the availability of more training data.

In summary, we introduced a multitask deep learning approach that enables the prediction of single-cell SGE from histology images by learning from H&E and SST images, and leveraging the relationships between multiple layers of cellular information. Our new GHIST framework outperformed current state-of-the-art SGE prediction methods based on spot-based technologies. Additionally, this framework offers a computational strategy to generate an in silico SGE platform with rich spatial information, enhancing existing databases. This facilitates the discovery of spatial associations between biomarkers, offering new insights into cellular biology and disease mechanisms.

## Methods

### Datasets and preprocessing

#### Subcellular in situ spatial transcriptomics data

(i) BreastCancer1 and BreastCancer2—The 10x Xenium breast cancer datasets included in this study were downloaded from https://www.10xgenomics.com/products/xenium-in-situ/preview-dataset-human-breast/ (accessed 14 March 2024). BreastCancer1 was characterized as T2N1M0, stage II-B, ER^+^/HER2^+^/PR^−^. BreastCancer2 was characterized as stage pT2 pN1a pMX, ER^−^/HER2^+^/PR^−^. The H&E image was aligned with its corresponding DAPI image for each dataset and visually verified. The overall pixel dimensions (*h* *×* *w*) of the H&E images were 25,778 × 35,416 for BreastCancer1, and 24,890 × 34,142 for BreastCancer2.
  Nuclei were segmented from the H&E images using Hover-Net^41^ that was pretrained on the CoNSeP dataset. The PyTorch weights of the segmentation-only model were downloaded from the official GitHub repository. The H&E WSIs were divided into nonoverlapping patches that were 3,000 × 3,000 pixels in size (or smaller patch sizes along the WSI border for the remaining pixels), and Hover-Net was applied to the patches. Nuclei detected from two adjacent patches that overlap along the patch borders were merged to form a single nucleus, to avoid division of a nucleus into more than one segment. Other segmentation algorithms including Otsu and StarDist^45^ were also compared, and best results were obtained using Hover-Net (Supplementary Fig. 18).
  BIDCell^46^ was applied to the transcripts and DAPI data to segment cells in the Xenium datasets and extract the gene expression within whole cells. Low-quality transcripts for Xenium data with a phred-scaled quality value score below 20 were removed, as suggested by the vendor^5^. Irrelevant data including negative control transcripts, blanks and antisense transcripts were filtered out. There were 313 unique genes for BreastCancer1 and 280 genes for BreastCancer2. For BIDCell, default parameter values from the exemplar file for Xenium and the provided single-cell reference file were used (both files were downloaded from the official BIDCell repository (https://github.com/SydneyBioX/BIDCell), version 1.0.3).
  To find the corresponding gene expression of cells detected from the subcellular SGE data and cells detected from H&E, the amount of overlap between nuclei from the two data types was computed. An H&E nucleus had a matching subcellular SGE nucleus if the amount of overlap between two corresponding nuclei was at least 50% of the area of the H&E nucleus. Cells with zero total expression counts, cells classified as ‘unassigned’ by scClassify and those with a nucleus smaller than 10 μm^2^ were filtered out. This resulted in a total of 94,000 cells with single-cell expression for BreastCancer1, and 80,000 cells for BreastCancer2. The count matrix output for the segmented cells from BIDCell was used to classify the cell types.
(ii) LungAdenocarcinoma—The 10x Xenium lung adenocarcinoma dataset with multimodal cell segmentation was downloaded from https://www.10xgenomics.com/datasets/preview-data-ffpe-human-lung-cancer-with-xenium-multimodal-cell-segmentation-1-standard/ (accessed 21 February 2024). The H&E image was aligned with its corresponding DAPI image for each dataset and visually verified. The overall pixel dimensions (*h* *×* *w*) of the H&E image were 17,098 × 51,187. There were 377 unique genes in this dataset.
  The same process used to segment nuclei from the H&E images for the breast cancer datasets was applied to the LungAdenocarcinoma dataset. We used the cell segmentation provided in the downloaded data bundle, which was achieved through the multimodal cell segmentation pipeline from 10x Genomics.
  The same process was used to determine the corresponding cells detected in H&E and subcellular SGE data and perform filtering of cells for the LungAdenocarcinoma dataset as the breast cancer datasets. For LungAdenocarcinoma, overlap was calculated with cells in the SGE data, rather than nuclei. This resulted in a total of 89,000 H&E cells with single-cell expression for LungAdenocarcinoma. The count matrix provided in the downloaded data bundle was used to classify the cell types.
(iii) Melanoma—The 10x Xenium melanoma dataset was downloaded from https://www.10xgenomics.com/datasets/human-skin-preview-data-xenium-human-skin-gene-expression-panel-add-on-1-standard/ (accessed 4 April 2024). The H&E image was aligned with its corresponding DAPI image for each dataset and visually verified. The overall pixel dimensions (*h* *×* *w*) of the H&E image were 27,276 × 31,262. There were 382 unique genes in this dataset.
(iv) BreastCancerILC and BreastCancerIDC—The 10x Xenium invasive lobular carcinoma (ILC) and invasive ductal carcinoma (IDC) datasets were downloaded from https://www.10xgenomics.com/datasets/ffpe-human-breast-with-pre-designed-panel-1-standard/ (accessed 22 January 2025). The overall pixel dimensions (*h* *×* *w*) of the H&E image were 41,405 × 48,511 for BreastCancerILC and 53,738 × 48,376 for BreastCancerIDC. There were 280 unique genes in these two datasets.
  The same data processing steps as for the other Xenium datasets were applied to the melanoma dataset. For this dataset, we used the gene expression within nuclei. After filtering, there were 47,000 H&E cells with single-cell expression. The count matrix for the segmented nuclei was used to classify the cell types.

#### scRNA-seq reference datasets used in cell-type classification

For each dataset, we used scClassify^47^ version 1.12.0, a supervised single-cell annotation tool, to annotate the cell types of each cell, which uses reference data with known cell types. We compared scClassify with other cell-type annotation tools including ScType^48^, SingleR^49^ and clustifyr^50^ and found scClassify produced the best result (Supplementary Fig. 19). The following scRNA-seq references are used for building the pretrained model for cell annotations.

- Single-cell breast reference—a publicly available breast cancer dataset from 10x Genomics (https://www.10xgenomics.com/products/xenium-in-situ/preview-dataset-human-breast/) as the reference to annotate the breast sample slides. The processing of the dataset is the same as detailed in our previous publication^46^. We compared the result using this reference with another breast cancer reference dataset^51^ that we obtained from the CZI CELLxGENE single-cell data portal (https://cellxgene.cziscience.com/collections/dea97145-f712-431c-a223-6b5f565f362a/). We compared two different settings using a broader cell-type category provided in the Wu et al. data that contains 7 cell types and a finer cell-type category provided in the Wu et al. data that contains 27 cell types. We named the data as Wu et al. major and Wu et al. minor, respectively. We found no noticeable differences compared to using the default reference data^46^ (Supplementary Fig. 20).
- Single-cell melanoma reference—we used the preprocessed melanoma dataset obtained from Durante et al.^52^ as it contains comprehensive annotations. We used the annotation provided, and grouped ‘class 1A primary tumor cells’, ‘class 2 PRAME^+^ primary tumor cells’, ‘class 1B PRAME^+^ Met tumor cells’ and ‘class 2 PRAME- primary tumor cells’ into ‘tumor’; ‘M2 macrophages’, ‘M1 macrophages’ and ‘monocyte’ into ‘macrophages’; ‘CD8, T effector memory’, ‘CD8, T resident memory’, ‘cytotoxic CD8’, ‘naive T cells’ and ‘regulatory T cells’ into ‘T cells’.
- Single-cell lung reference—we used the preprocessed lung atlas data, published by Sikkema et al.^53^, obtained from the CZI CELLxGENE data portal (https://cellxgene.cziscience.com/collections/6f6d381a-7701-4781-935c-db10d30de293/). It is one of the largest collections of single-cell lung data. We used the level 2 annotation in the ann_level_2 metadata column. We selected the main cell types: ‘blood vessels’, ‘airway epithelium’, ‘alveolar epithelium’, ‘fibroblast lineage’, ‘lymphatic endothelial cells’, ‘lymphoid’, ‘myeloid’ and ‘smooth muscle’. We grouped ‘airway epithelium’ and ‘alveolar epithelium’ into ‘epithelium’.

#### HER2ST spatial transcriptomics dataset

The HER2ST dataset^23^ was measured using SRT to investigate SGE in HER2^+^ breast tumors, from which the original investigators discovered shared gene signatures for immune and tumor processes. The dataset consists of 36 samples of HER2^+^ breast tissue sections from eight individuals.

For the histology images, 224 × 224-pixel patches were extracted around each sequencing spot. For GHIST, the patches were center-cropped at 112 × 112 pixels and resized to 256 × 256 pixels, so that the resolution is suitable for the nuclei segmentation model trained on NuCLS, and to be consistent with single-cell settings. For the SGE data of each tissue section, the top 1,000 HVGs for each section were considered and those genes that were expressed in less than 1,000 spots across all tissue sections were excluded. This resulted in 785 genes for the training of all models.

#### NuCLS datasets

The NuCLS dataset^54^ was used to provide cell segmentation and cell-type labels for the HER2ST dataset, to enable the training of GHIST to predict spot-level gene expression. The NuCLS dataset was downloaded from https://github.com/PathologyDataScience/BCSS/ (accessed 18 February 2024), and contains annotations of over 220,000 nuclei from breast cancer H&E images from TCGA. The annotations include a mixture of region-level (rectangular) masks and nuclei-level (outlining nuclei boundary) masks for individual nuclei. The annotations were created through a crowdsourcing approach involving pathologists, pathology residents and medical students. There were 1,744 H&E patches in the dataset. The ‘classes’ level of annotations were used as cell-type labels for nuclei. The classes include six cell types: ‘lymphocytes’, ‘macrophages’, ‘stromal cells’, ‘plasma cells’, ‘tumor cells’ and ‘other’.

The original nuclei masks provided in the NuCLS were unsuitable to be directly used for segmentation training, due to the presence of coarse rectangular nuclei masks. Hover-Net (pretrained on the CoNSeP dataset, weights downloaded from the official GitHub repository) was applied to the NuCLS H&E patches to generate fine-grained nuclei-level masks (as pseudo ground-truth masks). Due to inconsistencies in the sizes of H&E patches, four 270 × 270 patches were cropped from each image, from each corner of the original patch, and used as input to perform segmentation.

For every ground-truth nucleus, centroids of pseudo nuclei within the area of the ground-truth nucleus mask were found. The pseudo nucleus with the centroid closest by Euclidean distance to the centroid of the ground-truth nucleus was deemed the corresponding nucleus. The ground-truth cell-type label was assigned to the matched pseudo nucleus mask and used to train Hover-Net from scratch to perform simultaneous nuclei segmentation and predict the cell type of the six types in the labels. Training was performed with a random training split of 90% of available patches, and default model hyperparameters were applied. The model was trained for a total of 100 epochs; only decoders for the first 50 epochs, and all layers for another 50 epochs, per the default settings. The model from the final epoch was applied to the HER2ST data to generate labels to train GHIST.

#### TCGA-BRCA dataset

Data from TCGA-BRCA^55^ were used to evaluate model generalizability. This dataset was chosen as the predictions on these H&E images could be evaluated through the matched gene expression, survival data and other ‘omics’. TCGA-BRCA data were downloaded using the TCGAbiolinks package^56^ version 2.29.6.

RNA-seq data were obtained by the following query options: project = ‘TCGA-BRCA’, data.category = ‘transcriptome profiling’, data.type = ‘gene expression quantification’, experimental.strategy = ‘RNA-seq’ and workflow.type = ‘STAR - counts’. Histology images were obtained by the following query: project = ‘TCGA-BRCA’, data.category = ‘biospecimen’, data.type = ‘slide image’, and experimental.strategy = ‘diagnostic slide’. Clinical (metadata) with variables for defining breast cancer subtypes for samples were downloaded from the TCGAretriever package version 1.9.1. SCNAs were downloaded from UCSC Xena (Genomic Data Commons (GDC) TCGA Breast Cancer).

In our study, we mostly focused on the HER2^+^ breast cancer subtype from TCGA-BRCA, as this was the subtype of the data (BreastCancer2 and HER2ST) used to train the model and is of clinical interest due to its aggressive nature. We selected the HER2^+^ individuals based on a positive entry in the ‘lab_proc_her2_neu_immunohistochemistry_receptor_status’ metadata column^57^. We supplemented our analysis using the luminal breast cancer subtype, which are selected based on either a positive entry in ‘breast_carcinoma_estrogen_receptor_status’ with a positive entry in ‘breast_carcinoma_progesterone_receptor_status’ or a positive entry in ‘breast_carcinoma_estrogen_receptor_status’ with a negative entry in ‘breast_carcinoma_progesterone_receptor_status’.

Overall we considered 92 TCGA HER2^+^ samples and 461 TCGA individuals with the luminal subtype consisting of matched images, RNA-seq data, somatic copy number data and clinical data. For H&E processing, images were read using the OpenSlide-Python library, and nonoverlapping 256 × 256 patches were then cropped from the highest resolution level of the WSIs. We also compared different patch sizes and found 256 achieved the best results (Supplementary Fig. 21). The patches were filtered out if the sum of the RGB values were greater (or more white) than a patch with RGB (220, 220, 220). Nuclei were segmented from the patches using Hover-Net.

During inference, we found that it was necessary to apply normalization to the H&E images for both nuclei segmentation (Hover-Net) and single-cell gene expression prediction (GHIST). We found that the stain color affected the number of nuclei that Hover-Net predicted. The torchstain package (version 1.3.0)^58^ was used to apply the Macenko normalization method^59^ to each H&E patch. The reference was an unnormalized patch from a TCGA-BRCA H&E WSI for which Hover-Net performed well visually. Macenko stain normalization was also applied to the original TCGA H&E patches when input to GHIST. Here, the reference image was the whole H&E image from Xenium, downsampled such that 1 pixel = 10 μm.

#### Mixed DCIS cohort

Ductal carcinoma in situ (DCIS) and invasive breast cancer tissue from 44 females who were diagnosed with invasive breast cancer was used to construct duplicate tumor microarray data (Supplementary Table 4). The breast cancer tissue was obtained from the Australian Breast Cancer Tissue Bank. This tissue was obtained with informed consent from the donors and the use of these tissues for unspecified future research use received ethical and governance approval. Their use in this project was reviewed and approved by the Western Sydney Local Health District Human Research Ethics Committee (approval number 2019/ETH02688).

These cases are considered mixed DCIS because the patient was diagnosed with DCIS tissue and adjacent invasive breast cancer at the same time. Separate cores containing DCIS only and IDC only were both included on this tumor microarray. The histological status in each core and section was confirmed by an experienced specialist breast pathologist. These individuals were diagnosed between 2006 and 2010, and their ages ranged from 29 to 79 years at the time of diagnosis. It was the first breast disease event for 43 of the 44 patients. It was the second breast disease event for one patient whose prior breast cancer details are unknown. Having had prior breast cancer did not exclude a patient from eligibility for this cohort. All 44 individuals were diagnosed with invasive breast cancer, with DCIS also present. Twenty-nine of the individuals had a primary histologic diagnosis of invasive ductal carcinoma, one had a primary histologic diagnosis of apocrine carcinoma, and 14 were given a primary histologic diagnosis of infiltrating carcinoma not otherwise specified. One of the individuals had bilateral invasive carcinoma whereby both breasts were affected with infiltrating carcinoma not otherwise specified. Regarding the histopathological grade of the 44 invasive carcinomas, 2 tumors were classed grade 1, 15 were classed as grade 2 and 27 were classed as grade 3. Tumors had varying ER, PR and HER2 status.

### Spatially resolved single-cell Gene expression from HISTology

#### Overview of GHIST

GHIST is a deep learning framework that considers the relationships between multiple layers of biological information to predict spatially resolved gene expression within individual cells from visual information captured in H&E-stained histology images. GHIST uses two types of input data at inference: (i) H&E images, and (ii), optionally, average gene expression profiles of cell types from reference datasets, such as the Human Cell Atlas. During training, subcellular SGE data are used to quantify single-cell gene expression for the paired H&E image to enable the framework to learn relationships between H&E images and gene expression within cells. Once trained, the framework can infer single-cell SGE from H&E images without requiring SGE data.

A major innovation in developing GHIST is the harnessing of the complex relationships between the histological appearance of cells, gene expression within cells, cell type and composition of neighboring cell types. This multilayered approach enhances the capacity of the framework to better discern the SGE patterns reflected by H&E, thereby facilitating greater biological insights. During training, cell-type and neighborhood composition information are derived from subcellular SGE data, which form the labels to train GHIST to predict this information from H&E-stained images. Hence, their interrelationships become captured in the trained model. During inference, GHIST predicts this additional information from H&E-stained images and uses it to improve predicted gene expression. The primary task of GHIST is spatially resolved gene expression prediction within individual cells, and this is supported by three auxiliary helper prediction tasks (morphology, cell-type and neighborhood composition):

(A) Visual information that describes nuclei morphology and cell type. The visual appearance of tissues, cells and nuclei reflects underlying biological information. GHIST extracts information regarding nuclei morphology, texture and the appearance of the environment and neighboring cells from H&E images. This is learned through a backbone network that performs joint nuclei segmentation and classification. Nuclei-level and patch-level features from the backbone are combined. These features are fed into two auxiliary (cell-type and neighborhood composition) and primary prediction tasks downstream, so that these tasks receive a comprehensive embedding of information from H&E images.
  The backbone is parameterized by a set of learnable parameters *θ* of a deep learning segmentation model. The backbone predicts the probability of cell-type classes and background at each pixel. In this way, the model learns the relationships between pixels with morphology and cell type. We used the popular UNet 3+^60^ (without the deep supervision component) as the backbone of our framework to perform nuclei segmentation and classification. This backbone architecture may be swapped out for other segmentation architectures. The architecture comprised an encoding branch and decoding branch with five levels of feature scales, and incorporated full-scale skip connections that combined low-level details with high-level features across different scales (resolutions).
(B) Cell-type information. The level of expression of genes in a cell is directly related to its cell type, as different cell types are known to be associated with distinct expression profiles and different marker genes. GHIST harnesses the relationship between gene expression and cell type by carrying out cell-type prediction as an auxiliary task in its multitask framework. This serves three key purposes: (i) incentivizes the extraction of visual features that target cell type, which implicitly informs the gene expression profile of the cell; (ii) provides an alternative means of assessing and penalizing the predicted expression during training; and (iii) assists with the estimation of the composition of cell types within a local neighborhood (see ‘Neighborhood characteristics’ below). As a result, the framework can also better extract meaningful features that improve the prediction of gene expression.
  To achieve point (ii), during training, the predicted gene expression is used as input to another component in the framework to predict cell type. This allows the framework to learn single-cell expression values that reflect characteristic profiles that correspond to different cell types.
(C) Neighborhood characteristics. Cells are known to exhibit particular patterns of spatial organization in tumors, with different compositional patterns in local neighborhoods^61,62^. Thus, we incorporate a module that learns to estimate the neighborhood cell-type composition of a given input patch as a further type of auxiliary output prediction. This provides two key benefits: (i) ensures that the combination of low-level features from individual cells is coherent with the local neighborhood, and (ii) aids with discerning cell types that are visually challenging to distinguish in H&E images. As an example in breast cancer, B cell and T cell nuclei exhibit remarkably similar visual characteristics. B cells tend to be mistaken for T cells when the frequency of T cells is relatively dominant. These cell types often occur together in local neighborhoods. We leverage this knowledge in GHIST by using the estimated neighborhood composition to capture a more comprehensive view of the cellular environment in H&E images.
(D) Spatially resolved gene expression in individual cells. The integration of the three components above allows the main task, single-cell SGE prediction, to be facilitated by the different interrelated biological information. Once trained, GHIST predicts the expression of hundreds of genes for every nuclei detected in an H&E image, without requiring additional cell-type, neighborhood composition or SRT information as inputs. GHIST can leverage averaged gene expression profiles of various cell types from single-cell references of the target tissue type as an optional input (ablation results in Supplementary Figs. 1 and 2). The selection of the reference data is flexible, as GHIST does not require a matched single-cell reference for the same sample of interest, the cell types do not need to align with the cell types being predicted, multiple reference datasets may be used, and some reference datasets may contain different cell types.

#### Extraction of visual information

The input to the model is a cropped patch from the H&E WSIs, $x\in {{\mathfrak{R}}}^{h\times w\times 3}$, where *h* is the height of the patch and *w* is the width of the patch, and 3 corresponds to the RGB color channels. All the cells in an input patch are processed simultaneously during one forward pass, thereby allowing the model to flexibly support patches containing an arbitrary number of cells.

The backbone predicts an output of shape $[h,w,{n}_{{{\mathrm{CT}}}}+1]$, where each element represents the probability of each cell type or background class at each pixel. During training, the backbone is trained to carry out nuclei segmentation and cell-type classification by minimizing the loss function as given by equation (1):

1 $${L}_{\rm{Morph}}=-\frac{1}{N}\mathop{\sum }\limits_{i=1}^{N}\mathop{\sum }\limits_{j=1}^{M}{I}_{{ij}}\log ({p}_{{ij}}),$$

where *N* is the number of pixels, *M* is the number of classes (cell types, and background), ${I}_{{ij}}$ is a binary indicator of whether class *j* is the correct classification for pixel *i* (1 if true, 0 otherwise), and ${p}_{{ij}}$ is the predicted probability that pixel *i* belongs to class *j*. *M* is only relevant during training, and depends on the granularity of the cell types of interest. In our experiments, we found PCC of predicted SGE with ground truth to reduce for large *M* (‘Discussion’).

Nuclei-level features are extracted by multiplying nuclei masks for all valid nuclei in the input patch in an element-wise manner to the feature volumes produced by the first and last convolutional layers within the backbone architecture. The resulting features are concatenated along the feature dimension, and then the features within each nuclei are summed and normalized by the size of the nucleus in pixels. This is done to counter the variability in nuclei sizes. This yields a unique feature vector with a dimension of 384. Furthermore, patch-level features are calculated by averaging the same two feature volumes across the patch. Each nucleus-level feature vector is concatenated with the patch-level feature vector of its corresponding patch. This is then processed through two fully connected layers with ReLU activation, to generate the resulting final feature vector that describes the morphology of each nucleus ${x}_{{{\mathrm{nucleus}}}}\in {{\mathfrak{R}}}^{1\times 256}$. This describes nuclei-level and patch-level visual features that are relevant to nuclei morphology, cell type and the local neighborhood from H&E images.

#### Leveraging cell-type information

The cell-type prediction module uses the extracted visual features of each nucleus, ${x}_{\rm{nucleus}}$, to predict the cell type of the nucleus. The module comprises a fully connected layer (256-dimensional intermediate features), followed by ReLU activation, and another fully connected layer that predicts the probability of each cell type. The argmax function is applied to obtain the predicted classes. This module is trained by minimizing, as given by equation (2):

2 $${L}_{{{\mathrm{CT}}},{{\mathrm{class}}}}=-\frac{1}{C}\mathop{\sum }\limits_{i=1}^{C}\mathop{\sum }\limits_{j=1}^{M}{I}_{{ij}}\log \left({p}_{{ij}}\right),$$

where *C* is the number of cells (that is, nuclei), *M* is the number of classes (cell types), *I*_*ij*_ is a binary indicator of whether class *j* is the correct classification for cell *i* (1 if true, 0 otherwise) and ${p}_{{ij}}$ is the predicted probability that cell *i* belongs to class *j*.

We introduce another module with the same composition of layers as the cell-type prediction module, with three associated training losses, ${L}_{{{\mathrm{CT}}},{\rm{embed}}}$, ${L}_{{{\mathrm{CT}}},{\rm{logits}}}$ and ${L}_{{{\mathrm{CT}}},{\rm{expr}}}$ (defined below) that work in tandem. This module separately takes as input the predicted expression and ground-truth expression, and predicts the corresponding cell type for each set of input expression values.

- ${L}_{{{\mathrm{CT}}},{\rm{expr}}}$ is the cross-entropy loss (equation (2)), computed exclusively when the inputs are the predicted expression.
- ${L}_{{{\mathrm{CT}}},{\rm{embed}}}$ addresses the consistency of intermediate representations derived from the ground-truth and predicted input expression, and is written as shown in equation (3):
  3 $${L}_{{{\mathrm{CT}}},{\rm{embed}}}=\frac{1}{C}\mathop{\sum }\limits_{i=1}^{C}\left(1-\cos ({x}_{{{\mathrm{pr}}},i},{x}_{{{\mathrm{gt}}},i})\right),$$
  where ${x}_{{pr},i}$ is the embedding for the predicted (‘pr’) expression of cell *i*, and ${x}_{{{\mathrm{gt}}},i}$ is the embedding for the ground-truth (‘gt’) expression of the cell.
- ${L}_{{{\mathrm{CT}}},{\rm{logits}}}$ addresses the consistency between the predicted cell types derived from predicted and ground-truth gene expression for a given cell as shown in equation (4):

4 $${L}_{{{\mathrm{CT}}},{\rm{logits}}}=\frac{1}{C}\mathop{\sum }\limits_{i=1}^{C}{\left({p}_{{{\mathrm{pr}}},i}-{p}_{{{\mathrm{gt}}},i}\right)}^{2},$$

where ${p}_{{{\mathrm{pr}}},i}$ is the output cell-type logits given the predicted expression of cell *i*, and ${p}_{{{\mathrm{gt}}},i}$ is the output cell-type logits given the ground-truth expression of the cell.

Taken together, predicted single-cell expression is encouraged to resemble profiles that better correspond to cell types. GHIST can work without cell-type labels, but at a cost to performance (Supplementary Figs. 1 and 2). Contributions of *L*_Morph_ and *L*_*CT*, class_ are shown in Supplementary Fig. 22. Generally, *L*_Morph_ has a small value relative to the other losses and, therefore, a smaller contribution to performance.

#### Leveraging neighborhood characteristics

GHIST estimates the composition of cell types in each input H&E patch. This module comprises two fully connected layers with ReLU activation, with the last layer outputting the same number of elements as the number of cell types in the training data. Each value represents the proportion of cells of a particular cell type in a patch. SoftMax activation is used to ensure the compositions sum to 1 for each patch. The input to this module is the average of ${x}_{{fv},{\rm{nucleus}}}$ for all nuclei in a patch.

This neighborhood composition (NC) module is trained by minimizing ${L}_{{{\mathrm{NC}}},{\rm{est}}}$ and ${L}_{{{\mathrm{NC}}},{\rm{pr}}}$, calculated for a given patch defined as given by equation (5):

5 $${L}_{{{\mathrm{NC}}},{\rm{est}}}={D}_{{{\mathrm{KL}}}}\left({P}_{{{\mathrm{est}}}}{\rm{||}}Q\right)=\mathop{\sum }\limits_{j=1}^{M}{p}_{\rm{est}}\log \frac{{p}_{\rm{est}}(j)}{Q(j)},$$

where ${D}_{{KL}}$ is the Kullback–Leibler (KL) divergence between the cell-type compositions estimated from the neighborhood composition module ${P}_{\rm{est}}$, and the ground-truth compositions *Q*, and *j* ranges over all the cell types. Similarly, for a given patch, the loss associated with the neighborhood composition module given the auxiliary predicted cell types according to equation (6):

6 $${L}_{{{\mathrm{NC}}},{{\mathrm{pr}}}}={D}_{{{\mathrm{KL}}}}\left({P}_{{{\mathrm{CT}}}}{\rm{||}}Q\right)=\mathop{\sum }\limits_{j=1}^{M}{p}_{{{\mathrm{CT}}}}\log \frac{{p}_{{{\mathrm{CT}}}}(j)}{Q(j)},$$

where ${P}_{{{\mathrm{CT}}}}$ is the composition calculated by summing the predicted cell types (‘Leveraging cell-type information’) and dividing by the total number of cells in a given patch. Thus, further consistency is imposed between the different layers of biological information within the model.

The estimated neighborhood composition provides further utility in accounting for cell types that are difficult to discern from H&E images. For example, B cells and T cells have similar morphology, and thus the model becomes biased toward the dominant cell type (usually T cells), while ignoring the minority cell type (B cells). Based on this, during inference, we use the estimated compositions to recover missing cell types. For cell type *t* (for example, immune cell types including B cells, myeloid cells and T cells), per equation (7):

7 $${v}_{t}=\alpha {n}_{\rm{cells}}(\;{p}_{{{\rm{est,t}}}}-{p}_{{{\mathrm{CT}}},t})|{\varPhi }_{t}|,$$

where ${n}_{\rm{cells}}$ is the number of cells in the patch, ${\varPhi }_{t} \sim N({p}_{\rm{est,t}},1)$ is sampled from a normal distribution, and *α* is an adjustable hyperparameter to allow different recovery rates. We set *α* to 2 in our experiments for B, myeloid and T cell types, and T cells initially predicted with high confidence (probability above 0.6) were masked from this mechanism. These values were selected empirically, according to performance on the validation sets. Next, the logits of the initial predictions ${y{\prime} }_{{{\mathrm{CT}}},t}$ for each cell are revised as given by equation (8):

8 $${y{\prime} }_{{{\mathrm{NC}}},t}={v}_{t}+{y{\prime} }_{{{\mathrm{CT}}},t}.$$

The final cell type is found by applying argmax across the cell types being refined, while not affecting other cell types. To ensure consistency, the final expression values are taken from the corresponding cell-type-specific prediction module (‘Gene expression prediction’). Disease-specific knowledge may also be incorporated into this mechanism (equations (7) and (8)), for example, decreasing the relative proportion of epithelial cells to malignant cells in invasive breast cancer. We only demonstrate this mechanism on the breast cancer datasets; it was not applied for the melanoma or lung adenocarcinoma datasets.

#### Spatially resolved gene expression prediction for individual cells

GHIST predicts the expression value of every gene in each cell in the H&E image, $y{\prime} \in {{\mathfrak{R}}}^{{n}_{\rm{genes}}}$. The number of genes predicted varies according to the size of the gene panel in the available SST data. The datasets used in our study had panel sizes ranging from 280 to 382 genes.

The input to this module is the embedding vector ${x}_{\rm{nucleus}}\in {{\mathfrak{R}}}^{256}$ for all nuclei in a patch (with dimensions = 256), and if used, a set of averaged gene expression profiles of various cell types from single-cell references of the target tissue type $R\in {{\mathfrak{R}}}^{{n}_{\rm{AvgExp}}\times {n}_{\rm{genes}}}$. The input averaged profiles are optional (see ablation study results in Supplementary Figs. 1 and 2). The reference data do not need to be matched for the same sample of interest, and ${n}_{\rm{AvgExp}}$ can represent a flexible number and categories of cell types from a single or multiple reference datasets (Supplementary Fig. 20). A higher number of cell-type expression profiles provided may improve performance. *R* is kept the same during training and inference.

GHIST carries out a linear regression to predict *y*′ by first predicting $W\in {{\mathfrak{R}}}^{{n}_{{{\mathrm{ref}}}}}$ as given by equation (9):

9 $$W({x}_{\rm{nucleus}})=({\rm{ReLU}}({x}_{\rm{nucleus}}{W\;}_{r}^{(1)}+{b}_{r}^{(1)})){W}_{r}^{\;(2)}+{b}_{r}^{(2)},$$

followed by applying SoftMax along the ${n}_{\rm{AvgExp}}$ dimension, where ${W}_{r}^{\;(1)}\in {{\mathfrak{R}}}^{256\times 256},{b}_{r}^{(1)}\in {{\mathfrak{R}}}^{1\times 256},{W}_{r}^{\;(2)}\in {{\mathfrak{R}}}^{256\times {n}_{{{\mathrm{AvgExp}}}}}$ and ${b}_{r}^{(2)}\in {{\mathfrak{R}}}^{1\times {n}_{{{\mathrm{AvgExp}}}}}$. The ${S}_{j}$ is the expression value of gene *j* calculated by the weighted sum of profiles for each cell as given by equation (10):

10 $${S}_{j}=\mathop{\sum }\limits_{k=1}^{K}{W}_{k} {R}_{{kj}},$$

In the setting without using averaged expression profiles as an input is given by equation (11):

11 $$S\left({x}_{\rm{nucleus}}\right)=\left({\rm{ReLU}}\left({x}_{\rm{nucleus}}{W}_{e}^{\;\left(1\right)}+{b}_{e}^{\left(1\right)}\right)\right){W}_{e}^{\;\left(2\right)}+{b}_{e}^{\left(2\right)},$$

where ${W}_{e}^{\;(1)}\in {{\mathfrak{R}}}^{256\times 256},{b}_{e}^{(1)}\in {{\mathfrak{R}}}^{1\times 256},{W}_{e}^{\;(2)}\in {{\mathfrak{R}}}^{256\times {n}_{{{\mathrm{genes}}}}}$ and ${b}_{e}^{(2)}\in {{\mathfrak{R}}}^{1\times {n}_{{{\mathrm{genes}}}}}$. Next, information about the spatial neighborhood is incorporated using the estimated cell-type neighborhood composition ${p}_{{est}}\in {{\mathfrak{R}}}^{{n}_{{CT}}}$ of a patch. We use multi-head cross-attention (with eight heads), which are fundamental components in Transformers. For each cell, ${p}_{{est}}$ forms the query, while *S* forms the key and value.

A linear layer is applied to the output of the multi-head cross-attention module, resulting in $b\in {{\mathfrak{R}}}^{{n}_{{{\mathrm{genes}}}}}$. Finally, the predicted gene expression of each cell is computed as given by equation (12):

12 $${y}^{{\prime} }={\rm{ReLU}}\left(S+b\right),$$

The setting without using single-cell reference as an input is given by equation (13):

13 $${y}^{{\prime} }={\rm{ReLU}}\left(b\right),$$

For the spot-based prediction mode, *y*′ for all nuclei in a patch are summed (for weakly supervised learning) as given by equation (14):

14 $${y{\prime} }_{{{\mathrm{spot}}}}=\mathop{\sum }\limits_{i=1}^{C}{y{\prime} }_{i},$$

The training loss associated with gene expression in the default single-cell mode is given by equation (15):

15 $${L}_{{{\mathrm{GE}}}}=\frac{1}{C}\mathop{\sum }\limits_{i=1}^{C}{\left({y{\prime} }_{i}-{y}_{i}\right)}^{2},$$

where ${y}_{i}$ is the gene expression for cell *i* obtained from SST. The spot-based mode is given in equation (16):

16 $${L}_{\rm{GE}}=\frac{1}{S}\mathop{\sum }\limits_{i=1}^{S}{\left({y{\prime} }_{{\rm{spot}},{i}}-{y}_{i}\right)}^{2},$$

where ${y{\prime} }_{\rm{spot}}$ is the gene expression for spot *i*.

When the estimated neighborhood compositions are used to recover missing cell types, a copy of equations (9)–(13) is made for a specific cell type. This component predicts the gene expression of this cell type, ${y{\prime} }_{\rm{adj}}$, which is used for cells that are predicted as cell type *t* from equation (8). An additional loss, ${L}_{{{\mathrm{GE}}},{\rm{adj}}}$, is calculated to train this component for cell type *t* as given by equation (17):

17 $${L}_{{\mathrm{GE}},{\rm{{adj}}}}=\left\{\begin{array}{cc}\frac{1}{C}\mathop{\sum }\limits_{i=1}^{C}{(\;{y}_{{\rm{adj},}{i}}^{{\prime} }-{y}_{i})}^{2} & \,if\,{t}_{gt,i}\,=\,t\\ 0 &{\rm{otherwise}}\end{array}\right.$$

#### Model training

The model was trained by minimizing the total loss representing the sum of all the losses over *N* training patches as given by equation (18):

18 $$\begin{array}{l}\min\mathop{\sum }\limits_{n}^{N}\left[{L}_{\rm{Morph}}+{L}_{{{\mathrm{CT}}},{\rm{class}}}+{L}_{{\mathrm{{CT}}},{\rm{embed}}}+{L}_{{{\mathrm{CT}}},{\rm{logits}}}+{L}_{{{\mathrm{CT}}},{\rm{expr}}}\right.\\\left.+{L}_{{{\mathrm{NC}}},{\rm{est}}}+{L}_{{{\mathrm{NC}}},{\rm{pr}}}+\frac{1}{{N}_{{{\mathrm{CT}}}}}{L}_{{{\mathrm{GE}}},{\rm{adj}}}+{L}_{{{\mathrm{GE}}}}\right]\end{array}$$

Due to the relatively small value of ${L}_{{{\mathrm{CT}}},{\rm{embed}}}$ observed during training, it was empirically scaled by a factor of 100. ${L}_{{{\mathrm{GE}}},{\rm{adj}}}$ was inversely scaled by ${N}_{{{\mathrm{CT}}}}$, the number of cell types in the auxiliary prediction. There were no further weightings for the other losses, to improve generalizability and ensure that our losses were not fine-tuned to any particular datasets. We trained the model on varying amounts of data and observed maximum accuracy on the full dataset, as expected (Supplementary Fig. 23).

### Practical implementation of GHIST

Cross-validation was carried out for the Xenium and HER2ST datasets. The training set was used to train parameters of the model. The validation set was used to evaluate models during training to allow parameters to be tuned. Results presented in the paper were calculated using the predictions using the test set for each of the folds of the cross-validation. The same cross-validation splits were used for each model to ensure fair comparison.

Fivefold cross-validation was carried out for each Xenium dataset. The whole H&E image in each dataset was horizontally sectioned into five nonoverlapping portions, with each portion serving as the testing data for a fold. The remaining portions formed the training and validation sets, where the validation set was a section that was a tenth of the whole H&E in height. The training portion of a fold was divided into nonoverlapping 256 × 256 patches. During inference, patches were divided such that there was a 30-pixel overlap (approximately the width of the largest nuclei in the data). The final prediction for each distinct nucleus was based on the input image containing the largest area of that particular nucleus.

For the HER2ST dataset, the SGE prediction was evaluated using fourfold cross-validation. The dataset was split into four folds, with samples from the same patient belonging in the same fold. In each iteration of the cross-validation, two folds were used as a training set, one fold used as a validation set and the remaining fold used as a test set. The best model within each fold was then chosen based on the epoch that produced the best PCC in the validation set.

For GHIST, batch size was set to 8 during training and inference. The model is flexible regarding the number of nuclei present in the patch, and outputs predicted gene expression as ${n}_{\rm{{cells}},{\rm{total}}}\times {n}_{{\rm{genes}}}$. The model was trained end-to-end from scratch for 50 epochs. The saved checkpoints (per epoch) were applied to the validation sets, and selection of the top-performing checkpoint was based on the best average of the rank in F1 score (of the auxiliary cell-type classification prediction component) and PCC (of the predicted single-cell expression). The top-performing checkpoint was then applied to the test set of the fold.

Weights of the convolutional layers in GHIST were initialized using the method by He et al.^63^. Input images were standardized using the mean and standard deviation of the training patches, and gene expression was log-normalized. We used standard on-the-fly image data augmentation by randomly applying a flip (horizontal or vertical), rotation (of 90°, 180° or 270°) in the (*x*,*y*) plane. The order of training samples was randomized before training. We used the AdamW optimizer^64^ to minimize the sum of all losses at a fixed learning rate of 0.001, with a first-moment estimate of 0.9, second-moment estimate of 0.999 and weight decay of 0.0001.

We ran GHIST on a Linux system with a 24-GB NVIDIA GeForce RTX 4090 GPU, Intel Core i9-13900F CPU @ 5.60 GHz with 32 threads, and 32-GB RAM. Using this setup, training was completed within 10 h using BreastCancer2, which contained around 80,000 cells, 850 M pixels and 280 genes. VRAM usage was approximately 21 GB and RAM usage was approximately 10 GB during training. Inference with GHIST was completed after around 20 h on the 92 TCGA-BRCA WSIs (around 63 million cells in total).

#### Application to TCGA-BRCA

Disease-specific knowledge was incorporated during inference on the TCGA images, due to the disparity between the sample that the model was trained on (which comprised normal ducts, DCIS and invasive carcinoma), and those of the TCGA cohort (all invasive subtypes). Without fine-tuning, we set *α* to an arbitrary high value (10,000) and considering ${p}_{{{\mathrm{CT}}}}$ as 0 for malignant cell types (equations (7) and (8)). During inference on TCGA images, ensemble averaging was carried out using the model checkpoints from three folds that were trained on BreastCancer2.

### Performance evaluation

We used a multilevel evaluation strategy to comprehensively assess GHIST from multiple aspects (Supplementary Fig. 24).

#### Evaluation study 1: ablation study

We performed an ablation study to determine the contributions from each of the types of biological information in GHIST. We used BreastCancer2 for these experiments. Furthermore, in another set of experiments, we used BreastCancer2, BreastCancerILC and BreastCancerIDC for training and validation, with BreastCancer1 reserved for testing. We evaluated GHIST without neighborhood composition prediction, thereby impeding the ability to refine predicted gene expression for cells based on estimated neighborhood characteristics. We also investigated the effects of performing auxiliary cell-type prediction, by removing the ability of the model to leverage the relationships between gene expression and cell-type labels. Furthermore, instead of performing a linear regression of average cell-type profiles, we investigated the setting where gene expression is directly predicted as a straightforward regression.

We performed cell-type classification using scClassify with the breast cancer reference data, followed by comparison of cell-type distribution and cell-type proportion with the ground-truth data and the correlation of SVGs.

#### Evaluation study 2: comparison study with other spot-based methods

We describe the settings for other spot-based methods below, and more details may be found in our benchmarking work^15^:

(A) Settings used for other methods
  ST-Net^10^—ST-Net was implemented in Python and PyTorch using hyperparameter values as outlined in the original paper. The DenseNet-121 backbone used was pretrained on ImageNet. ST-Net was trained using the mean squared error (MSE) loss function, computed between predicted and ground-truth expression levels.
  HisToGene^9^—The HisToGene model was implemented using PyTorch with the following hyperparameters: a learning rate of 10 × 10^−5^, the number of training epochs of 100, a dropout ratio of 0.1, 8 multi-head attention layers and 16 attention heads. The final model used was the model after all epochs, per the HisToGene pipeline.
  GeneCodeR^13^—GeneCodeR was the only method that did not use deep learning for its model. The following hyperparameters were used for the coordinate descent updating algorithm: the dimensions of the encoding space for the samples (*i_dim*) of *k* = 500, the dimensions of the encoding space for the features (*j_dim*) of *c* = 500, initialization for the alpha_sample = ‘rnorm’, initialization for the beta_sample = ‘irlba’, maximum iterations of 20, tolerance of 5, learning rate of 0.1 and batch size of 500.
  DeepSpaCE^12^—The DeepSpaCE model was then implemented using the VGG16 model as the backbone with the following hyperparameters: maximum number of training epochs of 50 (with training stopping before 50 if there is no improvement within 5 epochs), a learning rate of 10 × 10^−4^ and a weight decay of 10 × 10^−4^.
  DeepPT^8^—DeepPT was implemented in Python and PyTorch using hyperparameter values as outlined in the original paper. The ResNet-50 backbone used was pretrained on ImageNet. DeepPT was trained using the MSE loss function, computed between predicted and ground-truth expression levels.
  Hist2ST^11^—The model was implemented using Python and PyTorch and ran with default parameters stated in the original paper. Some hyperparameter changes were made for benchmarking including: increased the image patch size from 112 × 112 to 224 × 224 and reduced the number of input and output channels in the depth-wise and point-wise convolutional blocks from 32 to 16 due to limited computational power. Image embedding dimension was increased from 1,024 to 2,048 as the original code of setting embedding size was hard coded based on the image patch size and the number of channels. The model was trained using a sum of MSE loss of predicted and ground-truth expression levels, ZINB and Distillation mechanisms.
  iStar^14^—The model was implemented in Python using PyTorch and trained for 400 epochs with a learning rate of 10^−5^, optimized via Adam. The input images were rescaled so that each pixel corresponded to 0.5 × 0.5 μm, ensuring that a 16 × 16-pixel patch represented an 8 × 8-μm² area, approximately the size of a single cell. Each 16 × 16 patch was embedded into a 579-dimensional space. MSE loss was used for the weakly supervised learning process.
  We used code from the official GitHub repositories for each method unless otherwise stated. We trained all the comparison methods from scratch using the same dataset and cross-validation splits. This was done for fair comparison, and because many of the methods did not provide weights of their trained models. We observed that the PCC of these methods is comparable between the scores obtained in our experiments and those reported in the original papers.

(B) Evaluation measures
  Concordance between predicted and measured gene expression—We followed the strategies described in our benchmarking work^15^ to assess the performance of GHIST on HER2ST data. Briefly, the evaluation methods used include PCC and SSIM. These metrics were first calculated at an image level (for example, correlation was measured for each gene per image), and then averaged over each individual, then averaged over each gene.
    HVGs and SVGs were deduced from the ground-truth SGE from the HER2ST dataset. The modelGeneVar function, followed by the getTopHVGs function with prop = 0.1 from the scran R package^65^ version 1.32.0 were used to obtain the HVGs in each ST dataset. For SVG selection, genes with adjusted *P* < 0.05 were identified as SVGs in each image sample in each dataset using the SPARK-X package in R^66^ (version a8b4bf2). For HER2^+^, only the SVGs that appear in more than 30 of 36 samples were selected, and correlation was calculated based on top 20 genes ranked by adjusted *P* value.
  Assessment of the translational potential with cross-validated *C*-index and Kaplan–Meier curves—We first selected the top 20 genes associated with the highest *C*-indices from the univariate Cox models built on the predicted SGEs for each gene. Next, a multivariate Cox regression was then built using these genes. Each model was evaluated by *C*-index and a log-rank *P* value based on the predicted SGE for each of the spot-based methods and GHIST, where *C*-index was calculated by comparing the ground-truth event times and events with model predictions for each patient within a test set.
    While many existing methods^67^ perform calculation of *C*-indices similarly to a resubstitution model, we have used a threefold cross-validation with 100 repeats. This meant that individuals were randomly split into three subsets. Each subset was used as a test set of individuals, while the rest of the patients were used to train models. Through repeating this process 100 times, we measured the *C*-indices for each test set to obtain an average *C*-index across 300 values. We also obtained the average prediction for each individual over all repetitions under cross-validation, which was then used to categorize individuals into high-risk and low-risk groups. Kaplan–Meier curves were then constructed for each of the high-risk and low-risk groups. The log-rank test was then used to obtain a *P* value to quantify the difference between survival of the two groups.

#### Evaluation study 3: performance evaluation at single-cell resolution

For visual assessment, we selected cell-type-specific marker genes using the FindMarkers function in Seurat to identify markers for each cell type. We visualized the predicted gene expression and the measured gene expression of the selected top marker genes.

For concordance between predicted and measured cell-type proportion, we predicted the cell types in the TCGA data using the single-cell breast reference as aforementioned. We then visually compared the cell types between predicted and measured intensities using bar plots and scatterplots.

For correlation between predicted and measured gene expression, we treated the measured gene expression from the SST data as the ground truth. We computed the PCC of a collection of genes between the predicted gene expression from H&E and the measured (ground truth) gene expression from SST. We calculated the PCC based on the (1) top 20 SVGs, (2) top 50 SVGs, (3) top 20 HVGs, (4) top 50 HVGs, (5) top 20 non-SVGs, (6) top 50 non-SVGs, (7) top 20 non-HVGs and (8) top 50 non-HVGs. We defined the top SVGs and HVGs using the default ordering of the genes from the SVG and HVG calculation. The non-SVGs and non-HVGs are defined as the genes ranked in the bottom of the SVG and HVG calculation. For example, the bottom 20 non-HVGs are defined to be the genes ranked in the bottom 20 of the HVG calculation, similarly for the bottom 20 non-SVGs.

The SVGs were calculated using Giotto rank^68^ version 1.1.2, one of the top-performing methods based on a recent benchmarking paper^69^. The HVGs were calculated based on the function FindVariableFeatures from the Seurat R package^70^ version 5.0.3. A list of all genes in each Xenium dataset and their corresponding SVG statistics is in Supplementary Data 1.

For assessment of the translational potential of spatial features, we examined whether the prediction expression contains information that can be used to stratify individuals into distinct survival groups. We used death as the survival event outcome, days to death as the survival time and days to last follow-up when there was no death event. We considered each of the feature types as described in ‘multi-view feature generation’ and Supplementary Table 5 and examined the discriminatory power of each feature type by estimating the high-risk and low-risk individuals based on a given set of features. For a given feature type, we selected the top 1% of the features, or the top five features, whichever number is greater, from each feature type separately (Supplementary Table 5) and used them to fit a Cox proportional hazard model (function crossValidate in the ClassifyR^71^ R package version 3.9.1). Next, for each of the feature types described in Supplementary Table 5, we used 5,000 repeated threefold cross-validation strategies to identify high-risk and low-risk individuals. In each iteration (one repeat), two folds of the individuals were used to train the Cox proportional hazard model based on the selected features, and individual risk was calculated on the remaining testing fold. This was repeated 5,000 times, resulting in a vector of a ‘risk score’ for each individual. For each individual, this resulted in a vector of 5,000 risks estimated when the individual was inside the ‘test set’ for each iteration. The final risk score was calculated as the median of this vector of risk estimates.

Using the final risk score, patients with a risk in the 0% to 25% quantile were defined as the ‘low-risk group’, while individuals in the 75% to 100% quantile were defined as the ‘high-risk group’. A Kaplan–Meier plot was then constructed, and a log-rank test was performed. The chi-square statistic and *P* value associated with the log-rank test were calculated to measure the difference in survival between the two groups. We used the chi-square statistics associated with each feature type to assess the discrimination capacity of each feature type. To ensure a fair comparison with TCGA, we first subset the TCGA bulk RNA-seq to the same set of genes as in the H&E gene panel.

### Downstream statistical and computational biology analysis

To examine the downstream application of GHIST, we applied GHIST trained on the dataset BreastCancer2 to predict the expression on 92 TCGA HER2 samples, 461 TCGA individuals with the luminal subtype and another breast cancer tissue microarray cohort (detailed in previous sections).

#### Multi-view feature generation

We generated multiple types of features based on the prediction gene expression to enable more comprehensive exploration of the TCGA individuals. Using the R package scFeatures^28^ version 1.4.0, we generated feature types from three distinct categories: single-cell-based cell-type proportion, single-cell-based cell-type-specific expression and spatial pattern. Each feature type contained tens to hundreds of features. All feature types are defined in the scFeatures^28^ publication. Supplementary Table 5 describes each feature type in detail.

#### Differential state analysis between conditions (ER^−^/PR^−^ versus ER^+^/PR^+^)

While we now have a computationally generated multi-sample, multi-group spatially resolved scRNA-seq TCGA HER2 dataset, we first focused on identifying markers for differential cell states for each cell type between individuals with ER^−^/PR^−^ versus ER^+^/PR^+^. We modeled this using a negative binomial generalized linear model implemented in the function pbDS in the R package muscat (version 1.18.0). Significant differential cell-state markers were defined as genes with log fold change > 1 and *P* value < 0.05. We visualized the expression of these cell-type-specific differential state genes as a heat map.

#### Unsupervised learning to detect new sub-cohort in ER^+^/PR^+^

Within the ER^+^/PR^+^ cohort, we performed *k*-means clustering based on all cell-type-specific differential state genes identified in the previous section to cluster into two groups. We used PCC as the similarity metric and used the default parameters for the rest. This was performed using the Heatmap function implemented in the ComplexHeatmap^72^ R package version 2.20.0.

The characteristics of the two resulting clusters were determined in three different ways:

- Survival analysis. We calculated the Kaplan–Meier curve for survival for cluster 1 and cluster 2 using the function survfit in R package survival version 3.6-4.
- Differential expression analysis. We generated a large collection of features for each individual in the TCGA data using scFeatures (‘Multi-view feature generation’). Next, we identified features with a differential based on a moderated *t*-test using the lmFit function implemented in the package limma (version 3.60.2). We visualized these results using volcano plots.
- Classification analysis. We further performed classification to examine the separability of the two subgroups based on various feature types. To avoid the potential issue of ‘double dipping’, we removed all differential state genes from the features in each feature type, as well as genes with a correlation above 0.7 with any of the differential state genes. We assessed the performance based on balanced accuracy using 100 repeated threefold cross-validation implemented in the crossValidate function in the R package ClassifyR. The crossValidate has an in-built feature selection using *t*-tests to select features with differential ‘measurement’ between cluster 1 and cluster 2. We used a support vector machine as the classification model backbone. For visualization of the most important features contributing to the classification, we selected top features based on frequency of inclusion, and visualized the values of these features in cluster 1 and cluster 2 samples using box plots.

#### *Trans*-acting associations between copy number aberration and nearest-neighbor correlation

Focal CNA was estimated using the GDC pipeline with the GISTIC^73,74^ method implemented and then thresholded to −1, 0 or 1, representing deletion, normal copy or amplification, respectively. Here we selected 91 TCGA HER2^+^ individuals and 458 individuals with the luminal subtype who had CNA results to perform downstream analysis, as a few individuals did not have CNA data. CNAs on chromosome X were removed. We first selected all genes that have both deletion and amplification with a frequency ≥ 5, which resulted in 2,177 CNA genes for HER2^+^ individuals and 15,608 genes for individuals in the luminal cohort. Next, for a given CNA_gene, we compared between two groups of individuals, one showing CNA_gene loss (−1) and another showing CNA_gene gain (+1). For these two groups, we identified differential patterning genes where spatial patterning was measured by nearest-neighbor correlation. This differential patterning analysis was repeated for all CNA_genes, and a matrix of *P* values representing the moderated *t*-tests for all CNA genes being tested against the 280-gene nearest-neighbor correlation was generated (Supplementary Table 2). We proposed a new metric quantifying the summation of −log_10_(*P*) in a genomic region and used this to present the number of CNAs that affect SGE. Each chromosome was split into genomic regions by a length of 1 Mb. Three hotspot regions were selected for highlighting differential nearest-neighbor correlation between gain and loss copy number by calculating −log_2_(fold change) in the HER2^+^ cohort.

### Reporting summary

Further information on research design is available in the Nature Portfolio Reporting Summary linked to this article.

## Online content

Any methods, additional references, Nature Portfolio reporting summaries, source data, extended data, supplementary information, acknowledgements, peer review information; details of author contributions and competing interests; and statements of data and code availability are available at 10.1038/s41592-025-02795-z.

## Supplementary information

Supplementary Information Supplementary Figs. 1–24.

Reporting Summary

Supplementary Tables 1–5 Supplementary Tables 1–5

Supplementary Data 1 Supplementary Data 1

## Source data

Source Data Fig. 2 Statistical source data.

Source Data Fig. 3 Statistical source data.

Source Data Fig. 4 Statistical source data.

Source Data Fig. 5 Statistical source data.

## Data Availability

All datasets used in this study are publicly available and were downloaded from the following links. 10x Genomics Xenium breast cancer samples 1 and 2, and single-cell data: https://www.10xgenomics.com/products/xenium-in-situ/preview-dataset-human-breast/; 10x Genomics Xenium lung adenocarcinoma: https://www.10xgenomics.com/datasets/preview-data-ffpe-human-lung-cancer-with-xenium-multimodal-cell-segmentation-1-standard/; 10x Genomics Xenium melanoma: https://www.10xgenomics.com/datasets/human-skin-preview-data-xenium-human-skin-gene-expression-panel-add-on-1-standard/; 10x Genomics Xenium breast cancer ILC and IDC: https://www.10xgenomics.com/datasets/ffpe-human-breast-with-pre-designed-panel-1-standard/; melanoma single-cell reference data was downloaded from dbGaP under accession code phs001861.v1.p1 (http://www.ncbi.nlm.nih.gov/projects/gap/cgi-bin/study.cgi?study_id=phs001861.v1.p1/). Additional breast cancer reference data from the CZI CELLxGENE data portal: https://cellxgene.cziscience.com/collections/dea97145-f712-431c-a223-6b5f565f362a/; lung atlas data from CZI CELLXGENE data portal: https://cellxgene.cziscience.com/collections/6f6d381a-7701-4781-935c-db10d30de293/; HER2ST dataset: https://github.com/almaan/her2st/; NuCLS dataset: https://github.com/PathologyDataScience/BCSS/. Regarding data for the DCIS cohort (approval from Australian Breast Cancer Tissue Bank), requests can be made to D.G.; TCGA-BRCA: https://portal.gdc.cancer.gov/projects/TCGA-BRCA/; TCGA-BRCA clinical data: https://www.cbioportal.org/study/clinicalData?id=brca_tcga/. Somatic copy number alteration for study name GDC TCGA Breast Cancer (BRCA) was downloaded from UCSC Xena via https://xena.ucsc.edu/. Default parameter values for BIDCell and single-cell reference files are available at: https://github.com/SydneyBioX/BIDCell/. Source data are provided with this paper.

## Code availability

Our code for GHIST is available via GitHub at https://github.com/SydneyBioX/GHIST/.
