# Supplementary material for: Spatial gene expression at single-cell resolution from histology using deep learning with GHIST
Source: Nat Methods. 2025 Sep 15;22(9):1900–10. doi: 10.1038/s41592-025-02795-z (PMC12446070; doi:10.1038/s41592-025-02795-z)
Supplement: Supplementary file 1 — Supplementary Figs. 1–24. [file 41592_2025_2795_MOESM1_ESM.pdf]

nature methods

Supplementary information https://doi.org/10.1038/s41592-025-02795-z

Spatial gene expression at single-cell resolution from histology using deep learning with GHIST

In the format provided by the authors and unedited

Supplementary Figures

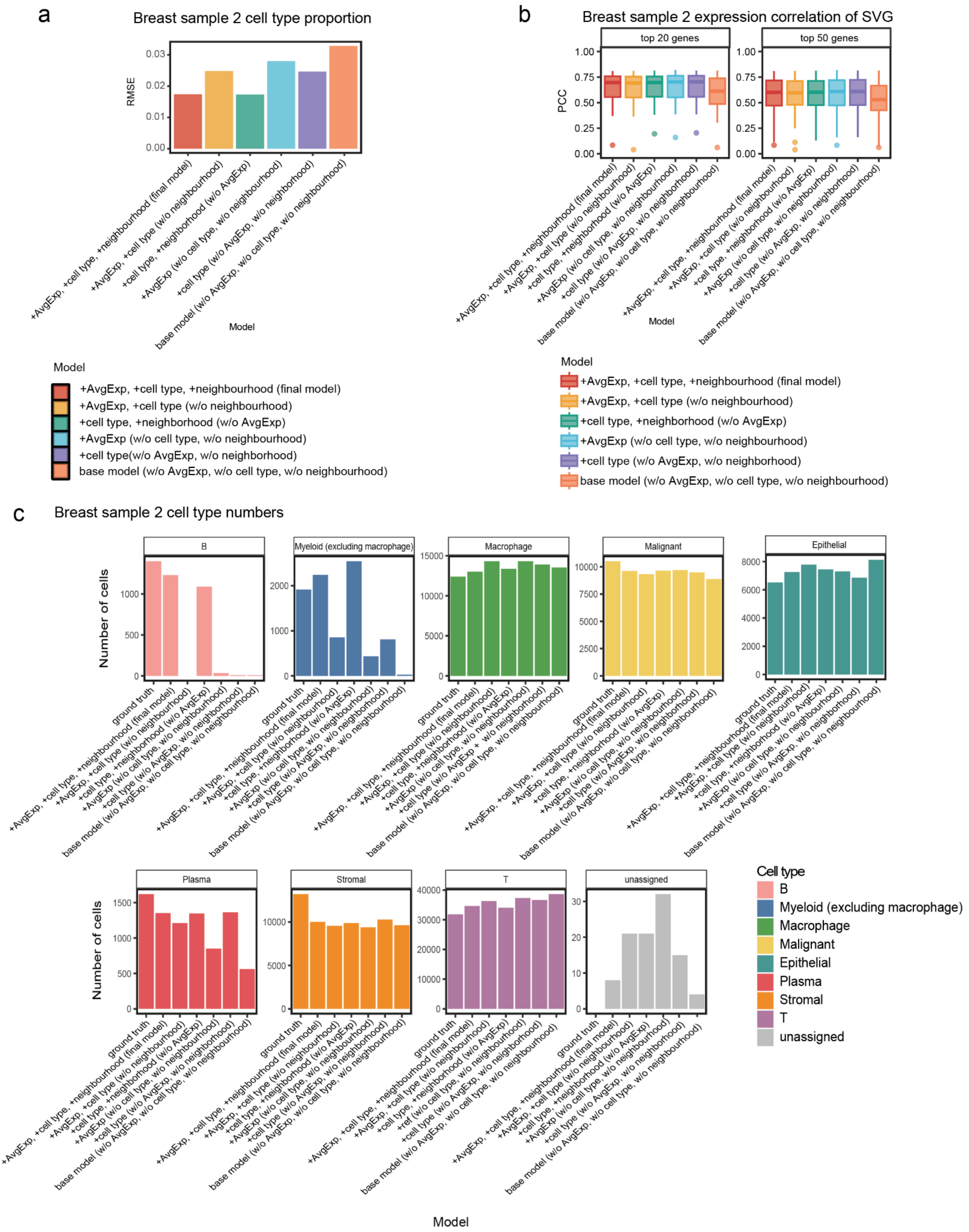

Supplementary Figure S1 Ablation study.
Performance of GHIST with and without key components (auxiliary cell type prediction, neighbourhood composition, and averaged cell type expression) on BreastCancer2. “w/o” stands for without the component, “+” represents the inclusion of the component, “AvgExp” stands for averaged cell type expression based on single-cell reference data, “celltype” stands for auxiliary

cell type prediction, “neighbourhood” stands for neighbourhood composition. (a) RMSE of the difference between predicted cell type proportion and ground truth cell type proportion. Cell types are predicted from scClassify using predicted single-cell expressions from the various settings mapped spatially across the slide. (b) PCC of predicted SVGs. PCC of the top 20 and top 50 SVGs were markedly lower in the base model. Each boxplot ranges from the first to third quartile with the median as the horizontal line. The lower whisker extends 1.5 times the interquartile range below the first quartile, while the upper whisker extends 1.5 times the interquartile range above the third quartile. The sample size corresponds to the number of genes included (either 20 or 50). (c) Number of cells for each predicted cell type. B and myeloid cells were missing in predictions without auxiliary cell type or neighbourhood composition prediction.

The integration of cell type and neighbourhood information leads to overall more accurate proportions of predicted cell types (as measured by RMSE between ground truth and predicted cell type proportions), and the ability to capture more cell types (in general) such as B and myeloid cells, which are lacking in other ablation settings. The integration of single-cell RNA-seq expression (“AvgExp”) improved PCC of predicted gene expression; and proportions of predicted cell types in the absence of cell type and neighbourhood information. This demonstrates the value of using single-cell RNA-seq expression, as cell type and neighbourhood information may not always be available during training. We do note that, in the situation where both average expression and cell type information are available, the average expression can be proxied from examining the cell type information and the spatial omics data jointly. We also note that when assessing the overall performance of the model, it is important to assess other measures in addition to correlation of gene expression including cell type measures and translational capacity, which cannot be fully captured by ablation studies alone.

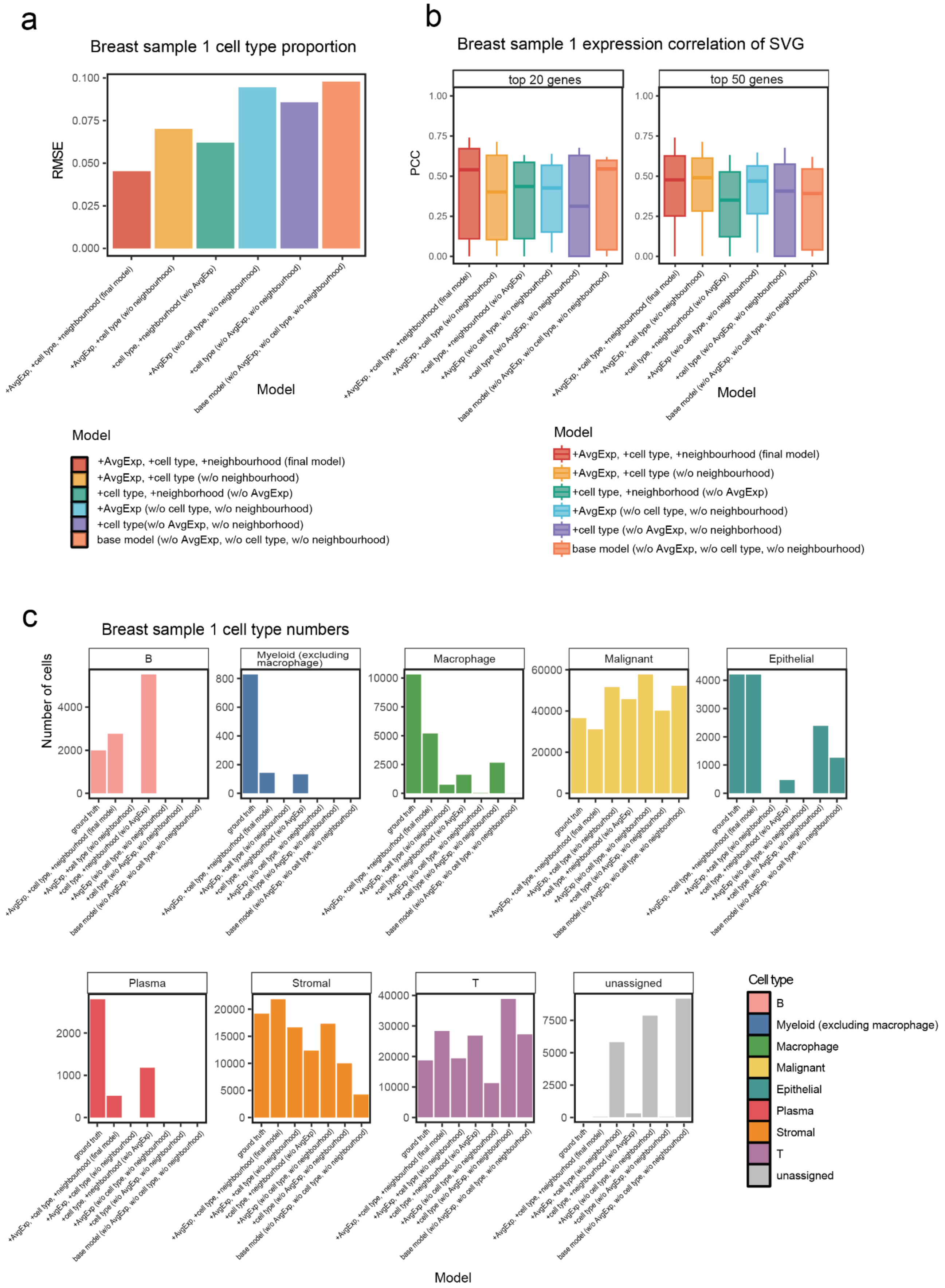

Supplementary Figure S2 Ablation study (cross dataset).
Performance of GHIST with and without key components (auxiliary cell type prediction, neighbourhood composition, and averaged cell type expression) tested on BreastCancer1, trained on combined data from BreastCancer2, BreastCancerILC, and BreastCancerIDC. “w/o” stands for

without the component, “+” represents the inclusion of the component, “AvgExp” stands for averaged cell type expression based on single-cell reference data, “celltype” stands for auxiliary cell type prediction, “neighbourhood” stands for neighbourhood composition. (a) RMSE of the difference between predicted cell type proportion and ground truth cell type proportion. Cell types are predicted from scClassify using predicted single-cell expressions from the various settings mapped spatially across the slide. (b) PCC of predicted SVGs. PCC of the top 20 and top 50 SVGs were markedly lower in the base model. Each boxplot ranges from the first to third quartile with the median as the horizontal line. The lower whisker extends 1.5 times the interquartile range below the first quartile, while the upper whisker extends 1.5 times the interquartile range above the third quartile. The sample size corresponds to the number of genes included (either 20 or 50). (c) Number of cells for each predicted cell type.

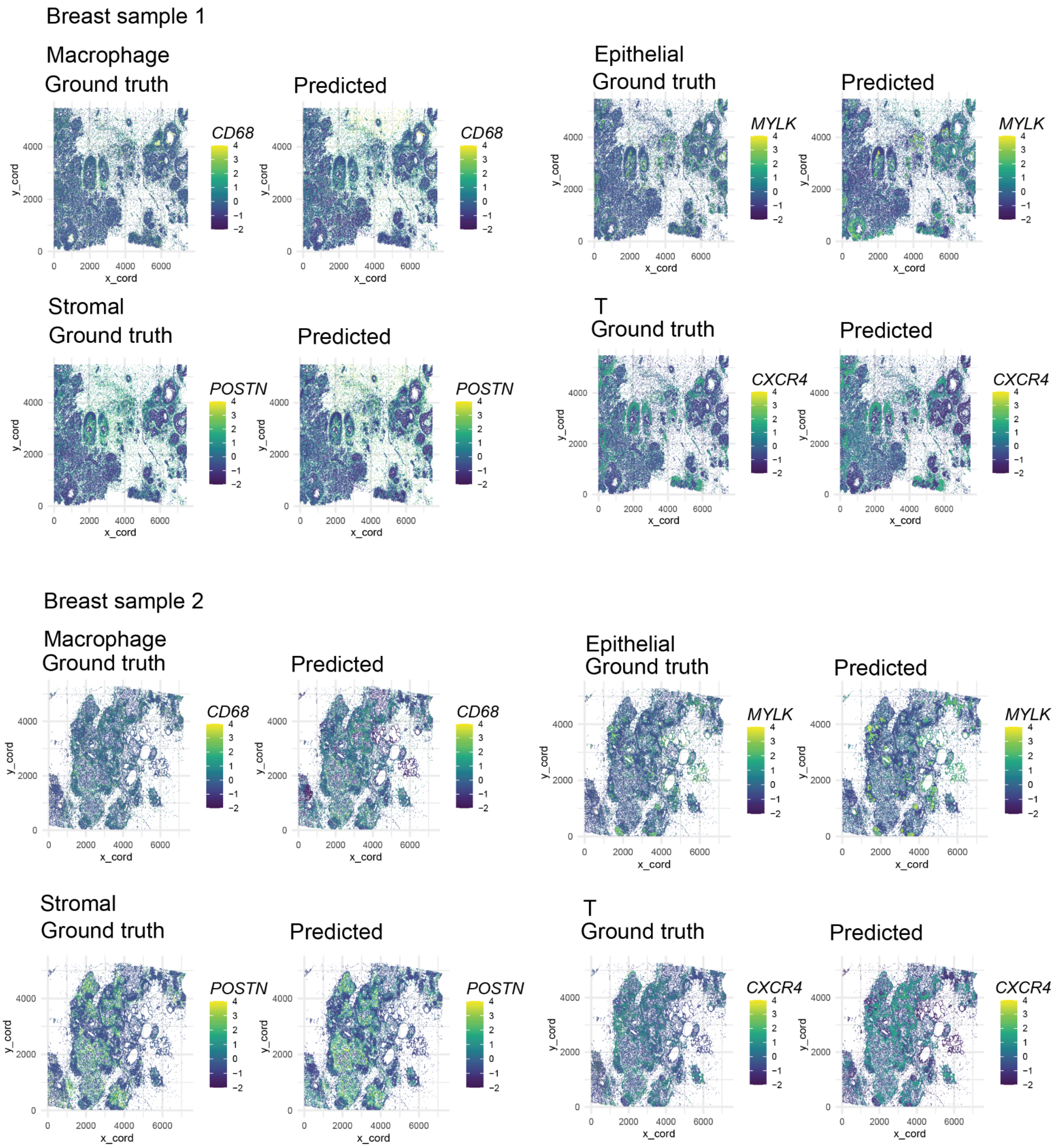

Supplementary Figure S3 Additional markers of different cell types.
Expression of predicted cell type markers (*CD68* for macrophages, *MYLK* for epithelial cells, *POSTN* for stromal cells, and *CXCR4* for T cells) mapped across the BreastCancer1 and BreastCancer2 slides.

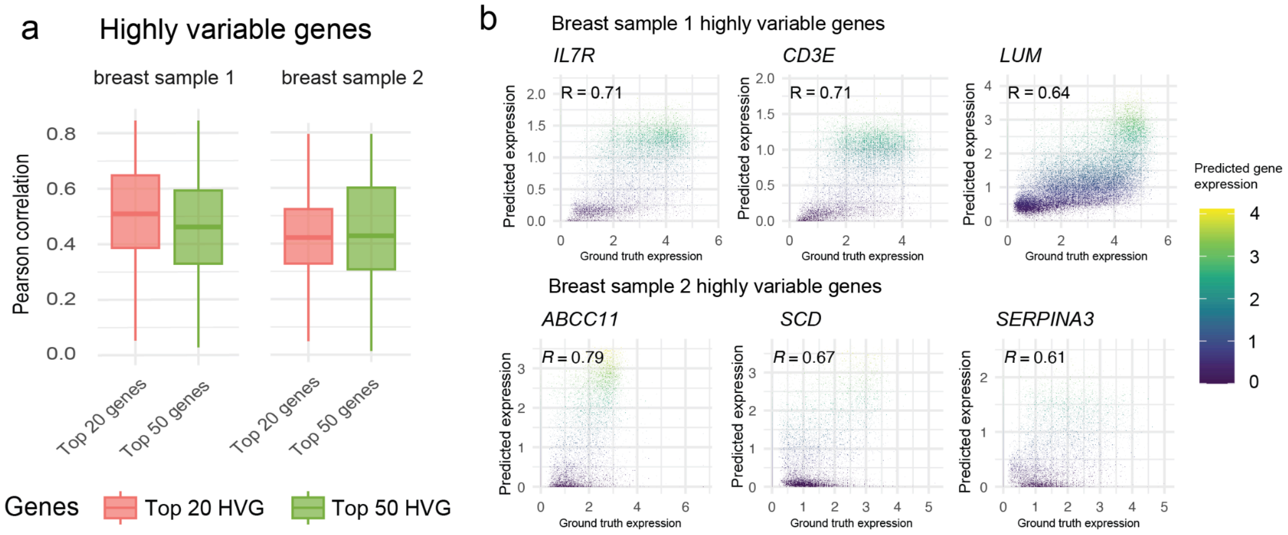

Supplementary Figure S4 PCC of predicted HVGs in the two breast cancer Xenium samples. (a) Boxplots of the PCC for the top 20 and 50 highly variable genes between the ground truth and predicted expressions. Each boxplot ranges from the first to third quartile with the median as the horizontal line. The lower whisker extends 1.5 times the interquartile range below the first quartile, while the upper whisker extends 1.5 times the interquartile range above the third quartile. The sample size corresponds to the number of genes included (either 20 or 50). **(b)** Scatter plot between the ground truth and predicted expressions for selected HVGs (*IL7R, CD3E, LUM, ABCC11, SCD*, and *SERPINA3*) in BreastCancer1.

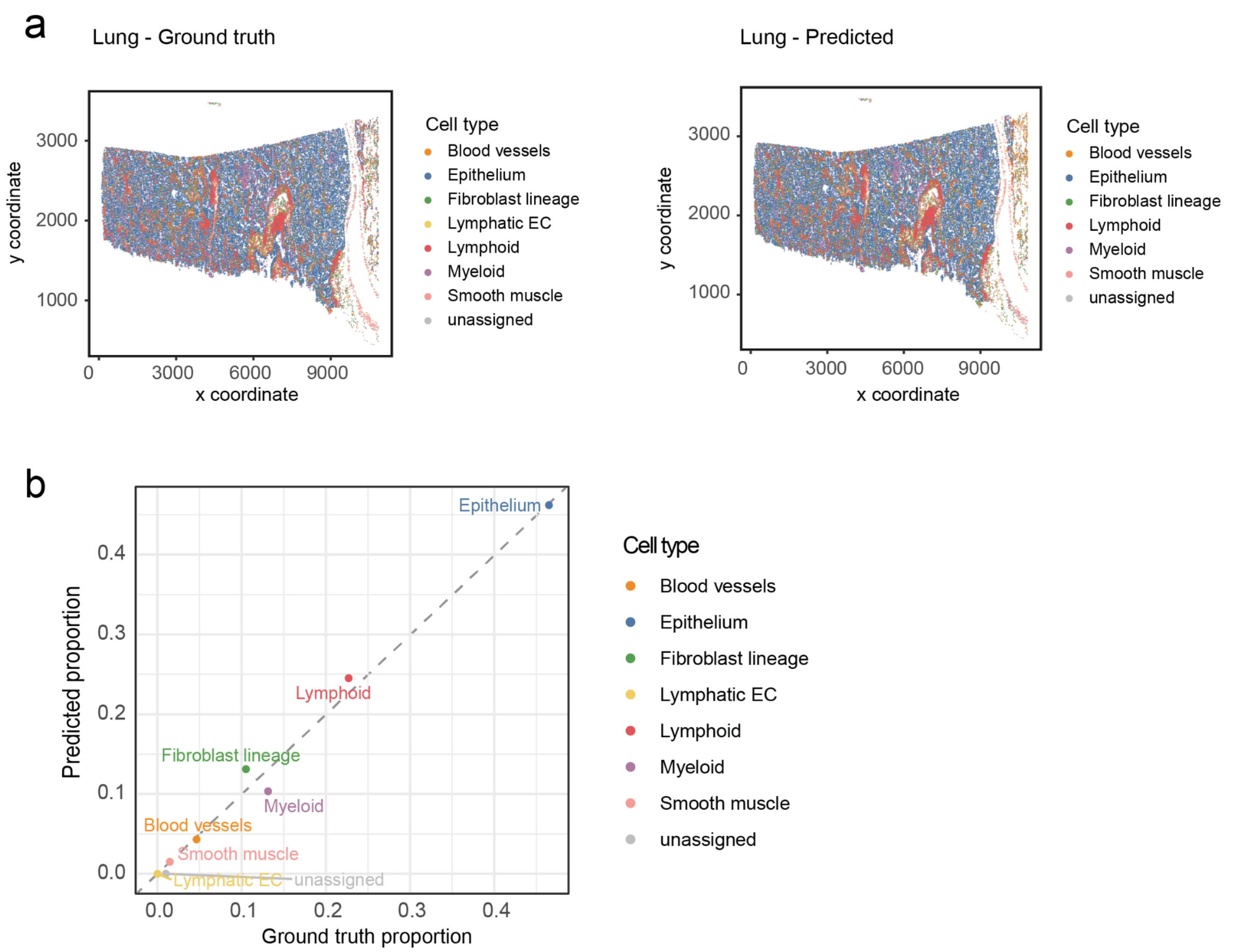

Supplementary Figure S5 LungAdenocarcinoma results.

(a) Cell types including blood vessels, epithelium, fibroblast lineage, lymphatic EC, lymphoid, myeloid, smooth muscle, and unassigned cells are mapped spatially across the tissue slide for the measured (ground truth) and predicted expressions. **(b)** Scatter plot between the measured (ground truth) and predicted cell type proportions.

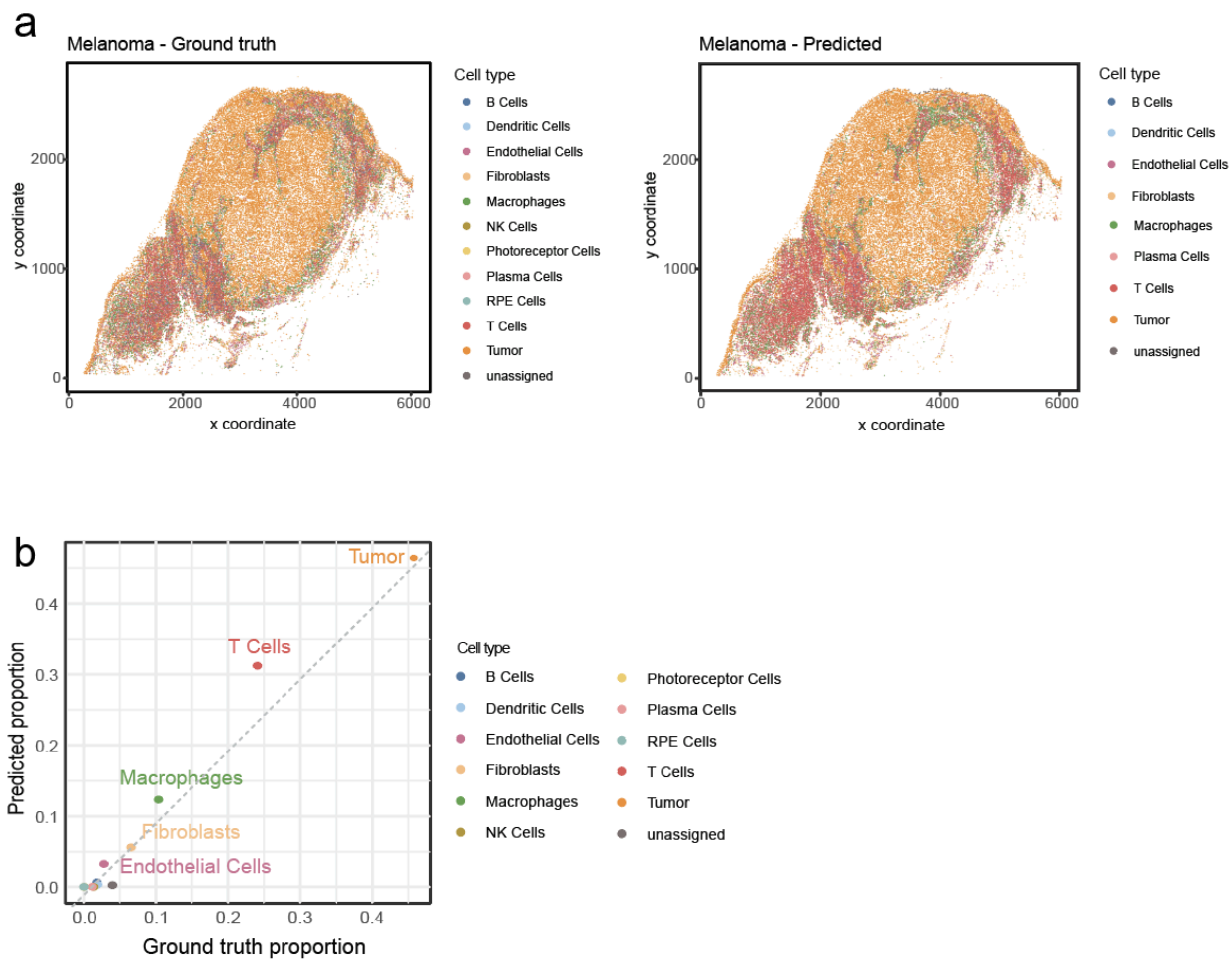

Supplementary Figure S6 Melanoma results.
(a) Cell types including B, dendritic, endothelial, fibroblasts, macrophages, NK, photoreceptor, plasma, RPE, T, tumour, and unassigned cells. These are mapped spatially across the tissue slide for the measured (ground truth) and predicted expressions. **(b)** Scatter plot between the measured (ground truth) and predicted cell type proportions.

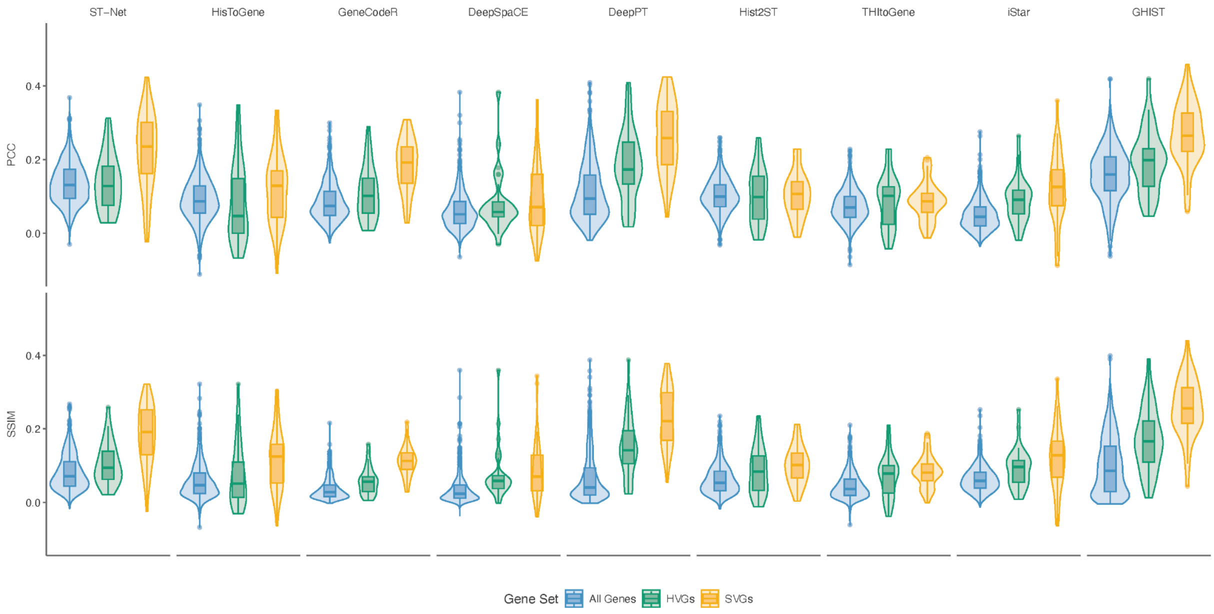

Supplementary Figure S7 Spot-based gene prediction and survival analysis performance among state-of-the-art methods and GHIST using the HER2ST dataset.
PCC and SSIM violin plots for each method for all genes (n = 785) as well as for selected HVGs (n = 30) and SVGs (n = 20 per image sample), demonstrating that selecting such types of genes is more biologically meaningful for comparison than all genes. Each plot ranges from the first to third quartile with the median as the horizontal line. The lower whisker extends 1.5 times the interquartile range below the first quartile, while the upper whisker extends 1.5 times the interquartile range above the third quartile.

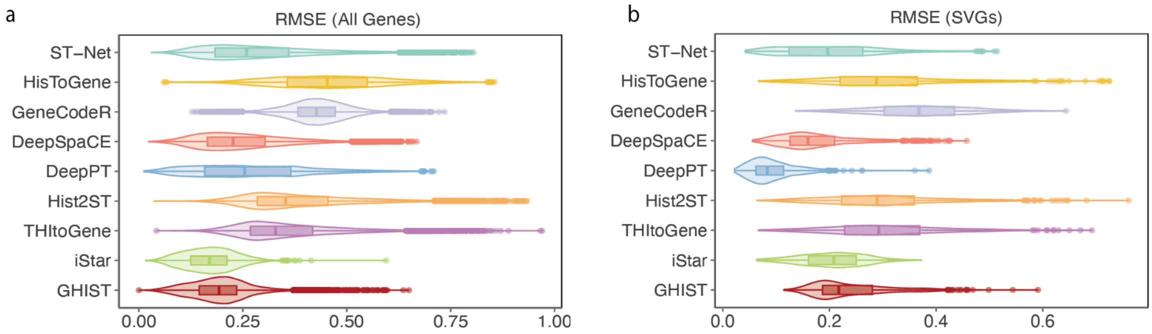

Supplementary Figure S8 Spot-based gene prediction RMSE among state-of-the-art methods and GHIST using the HER2ST dataset.
Violin and boxplots of RMSE between ground truth gene expression and predicted gene expression of **(a)** all genes (n = 785) and **(b)** SVGs (n = 20 per image sample). Metrics measured from the test fold of a 4-fold CV, averaged over each gene across the HER2ST dataset. Each boxplot ranges from the first to third quartile with the median as the horizontal line. The lower whisker extends 1.5 times the interquartile range below the first quartile, while the upper whisker extends 1.5 times the interquartile range above the third quartile.

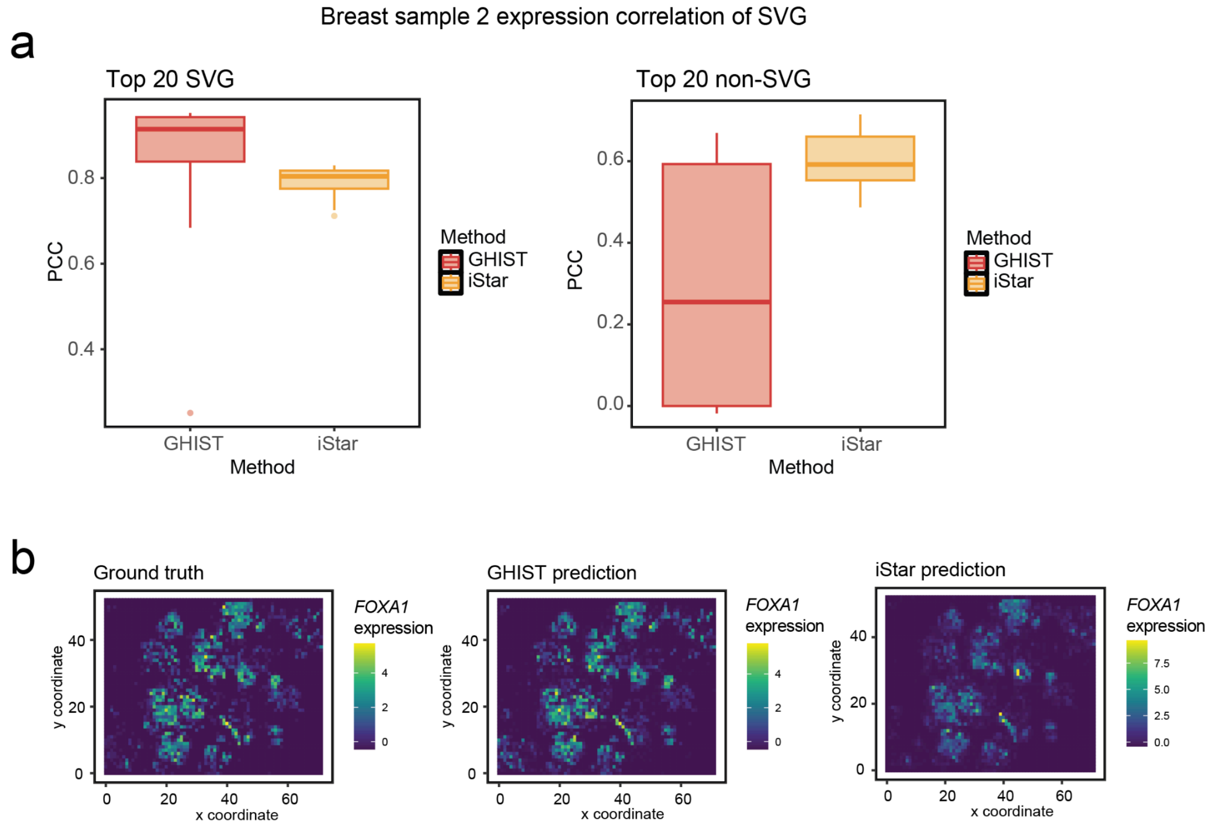

Supplementary Figure S9 Performance comparison between GHIST and iStar using BreastCancer2 (pseudo-Visium).
(a) PCC of the top 20 SVGs (left) and top 20 non-SVGs (right). The sample size corresponds to the number of genes included, i.e., 20. (b) Comparison of predicted expression for *FOXA1*.

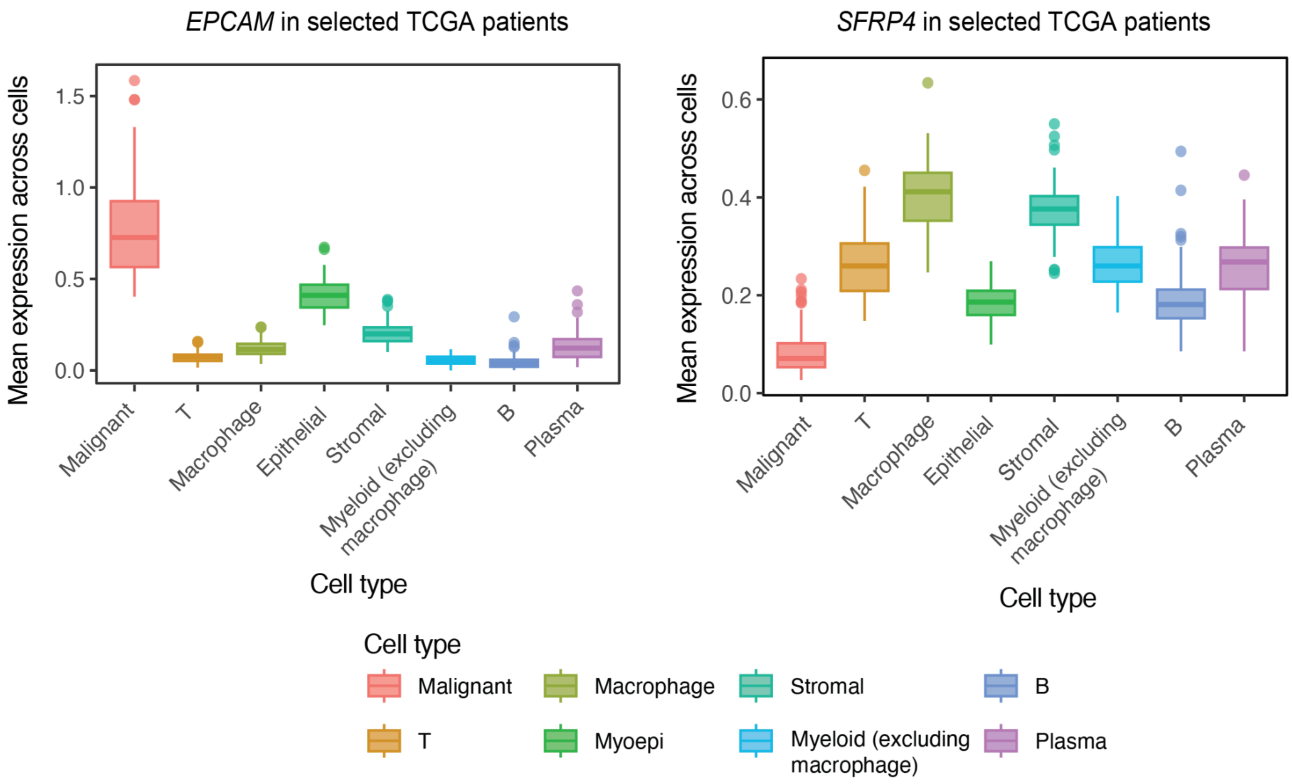

Supplementary Figure S10 Predicted expression of selected genes in different cell types. Box plots showing the mean predicted expression of *EPCAM* and *SFRP4* across cells of different cell types in the TCGA patients (n = 92). Each boxplot ranges from the first to third quartile with the median as the horizontal line. The lower whisker extends 1.5 times the interquartile range below the first quartile, while the upper whisker extends 1.5 times the interquartile range above the third quartile.

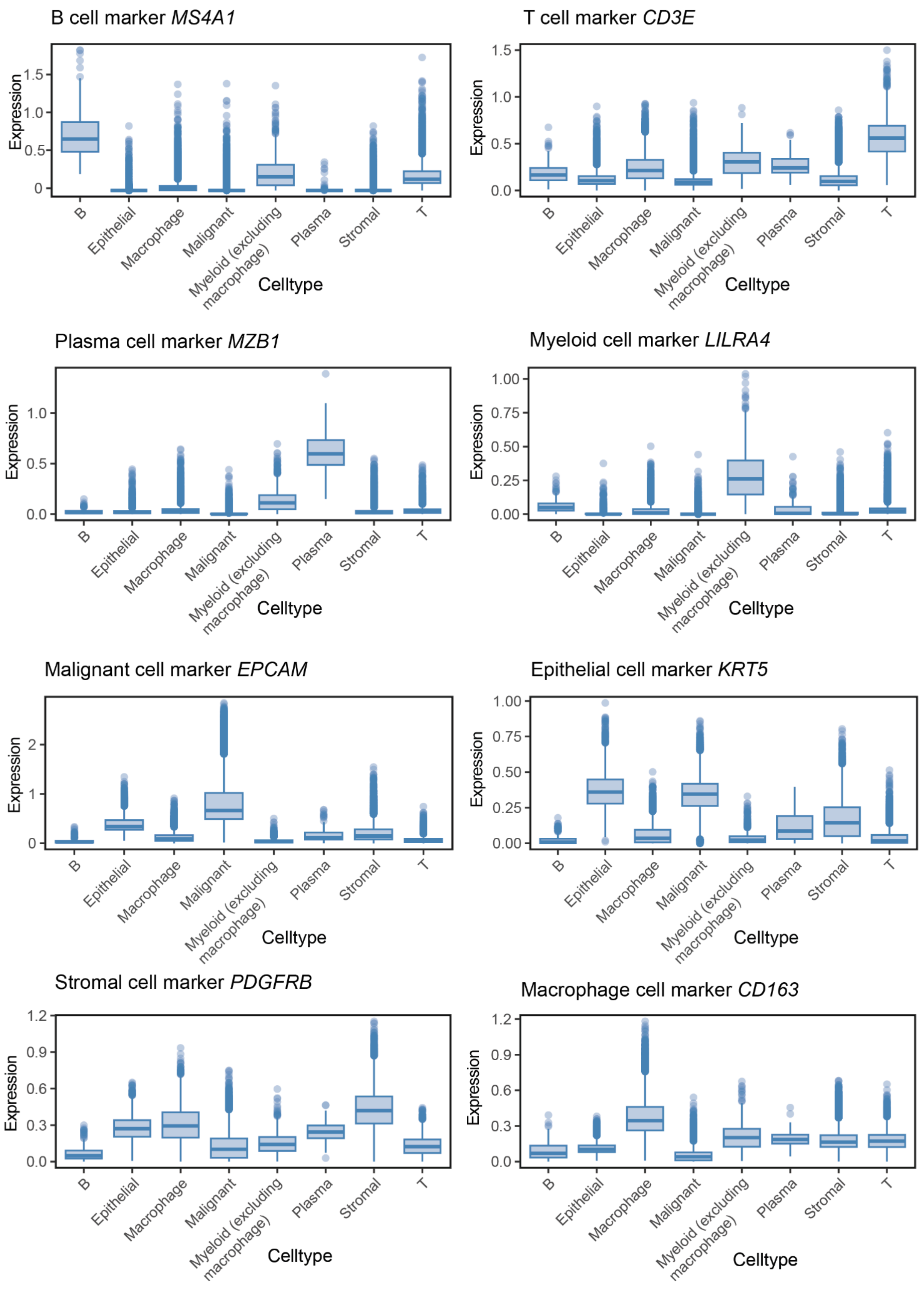

Supplementary Figure S11 Expression of marker genes.

Boxplots show the expression distribution of selected marker genes for each cell type predicted in the 92 TCGA HER2+ breast cancer patients (n = 92). Each boxplot ranges from the first to third quartile with the median as the horizontal line. The lower whisker extends 1.5 times the interquartile range below the first quartile, while the upper whisker extends 1.5 times the interquartile range above the third quartile.

a b

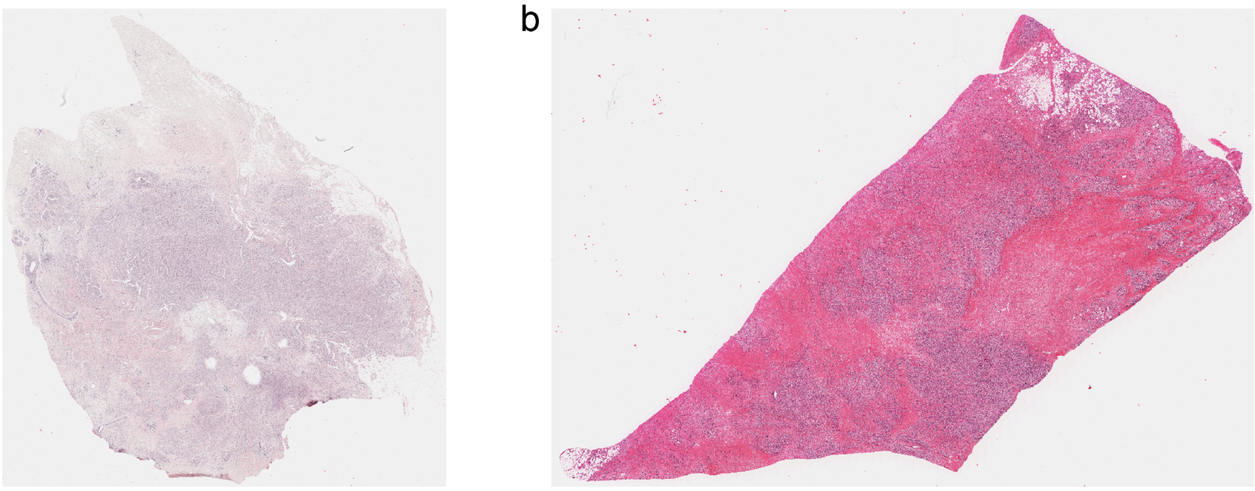

Supplementary Figure S12 Stain quality issues in TCGA-BRCA.
Examples of issues with stain quality observed in the H&E images from TCGA-BRCA patients, where **(a)** TCGA-AO-A12G appeared understained and faded, and **(b)** TCGA-PE-A5DD appeared very overstained.

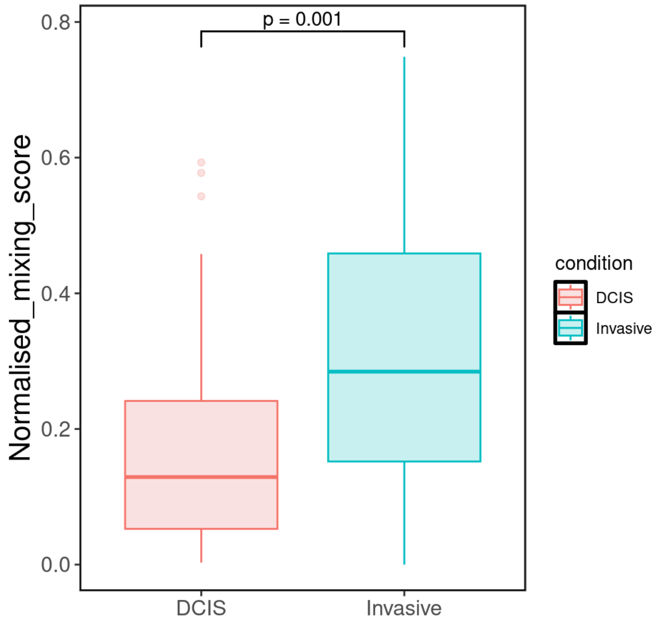

Supplementary Figure S13 Normalised mixing score between DCIS and invasive region. We used in-house breast cancer tissue data that contain both in situ and adjacent invasive tumour cores. Using this data, we characterised tumour-immune microenvironment changes during DCIS-invasive transition. To quantify the degree of immune infiltration in tumour regions, we used normalised mixing score[74], which is defined as the proportion of immune cells touching tumour cells and was formally calculated as the number of immune-tumour interactions divided by the number of immune-immune interactions, accounted for the total number of cells in the tumour and immune population was also used for comparison. Linear mixed model was fitted to compare normalised mixing score between the two stages (n = 44) and two-sided *P* value was reported. No multiple comparison adjustment was required. Each boxplot ranges from the first to third quartile with the median as the horizontal line. The lower whisker extends 1.5 times the interquartile range below the first quartile, while the upper whisker extends 1.5 times the interquartile range above the third quartile.

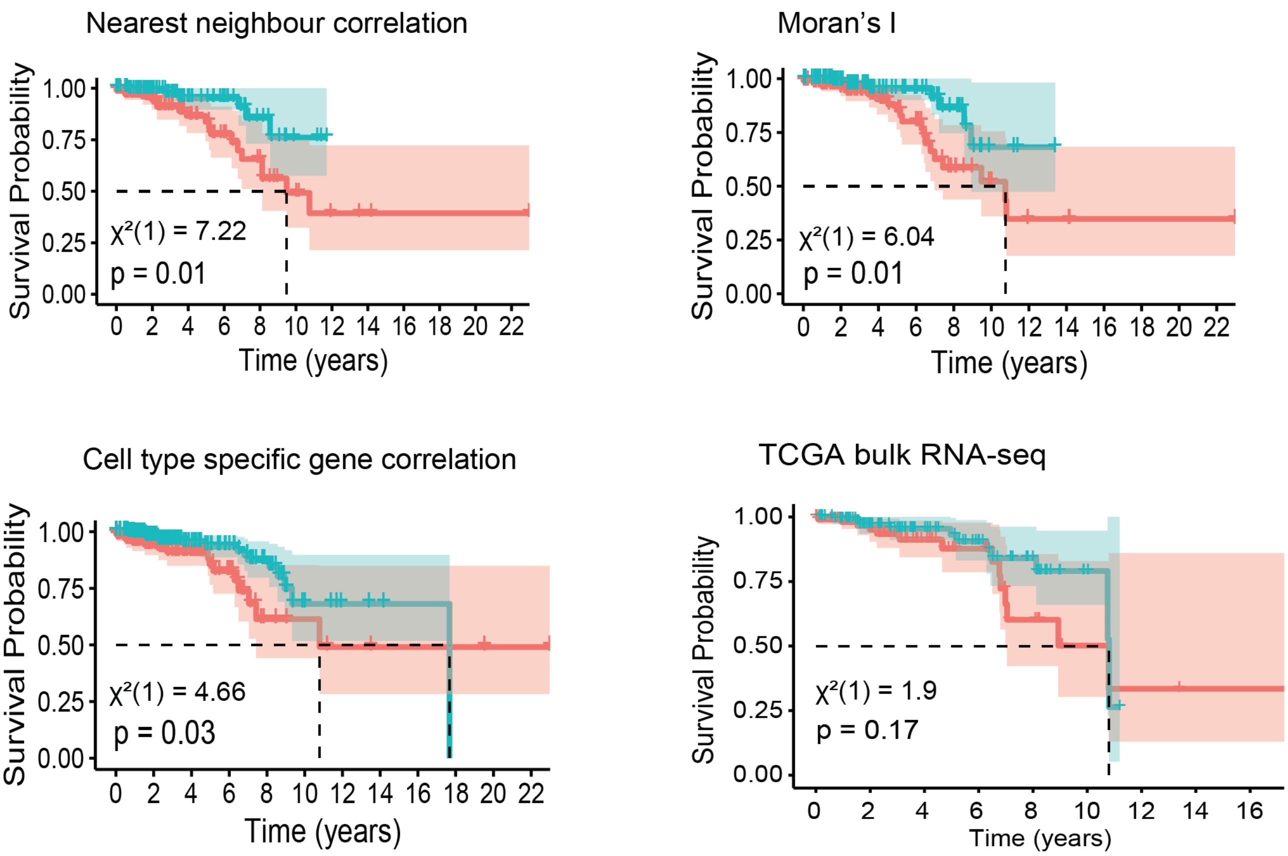

Supplementary Figure S14 KM curves for the TCGA breast cancer luminal subset using various features types from scFeatures.

Nearest neighbour correlation represents the correlation of gene expression with neighbouring cells. Moran’s I is a spatial autocorrelation metric. Cell type specific gene correlation is the correlation of pairwise gene expression within each cell type. Shaded regions represent 95% confidence intervals. A two-sided log-rank test was used to calculate the χ^2 (chi-squared) test statistic and *P* value of the survival difference between the two groups (n = 92).

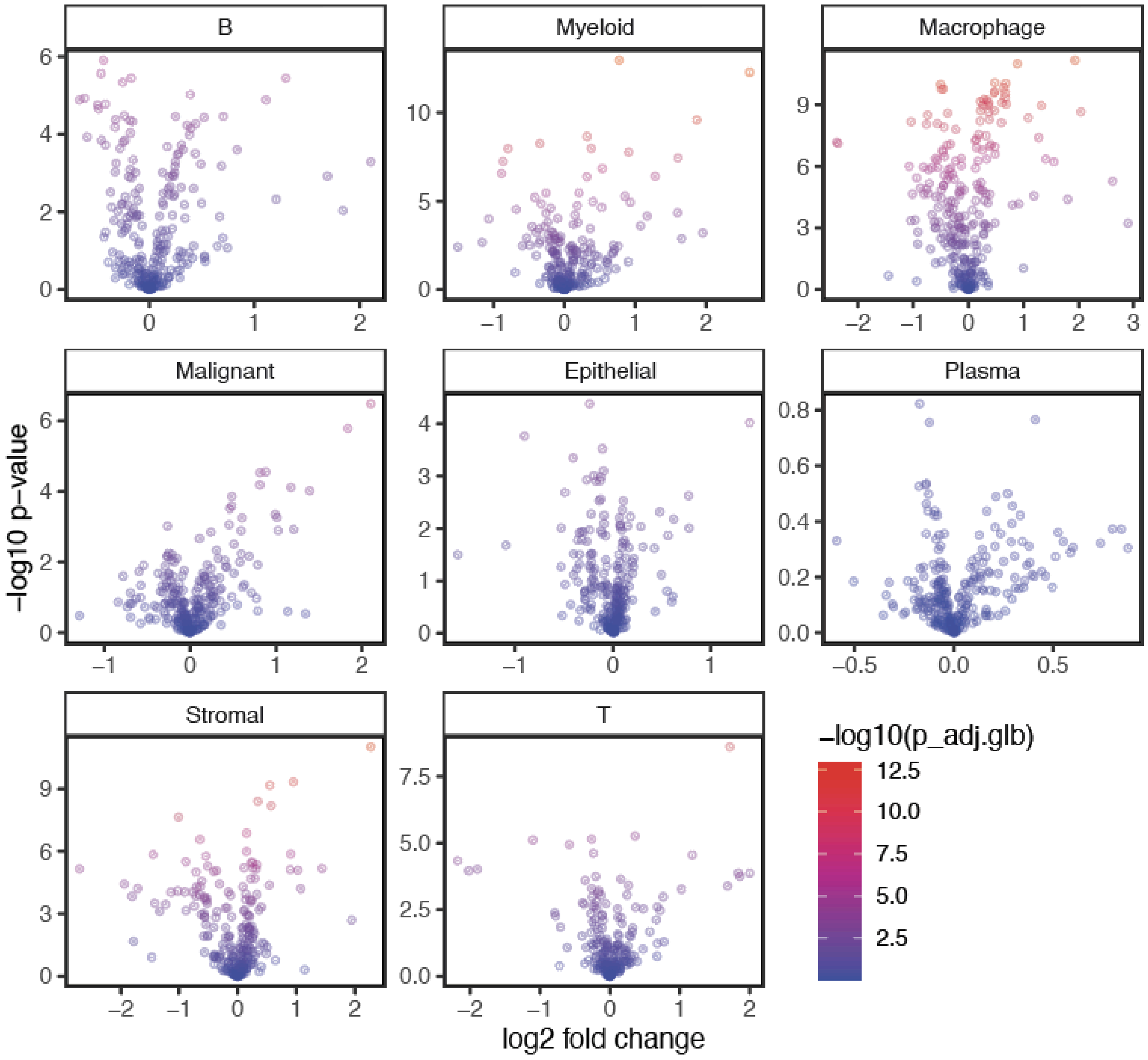

Supplementary Figure S15 Volcano plots between Cluster 1 and Cluster 2 split by different cell types.

Volcano plot showing significant differentially expressed genes between the cluster 1 and cluster 2 refined $ER^{+/}PR^{+}$ patients (n = 54).

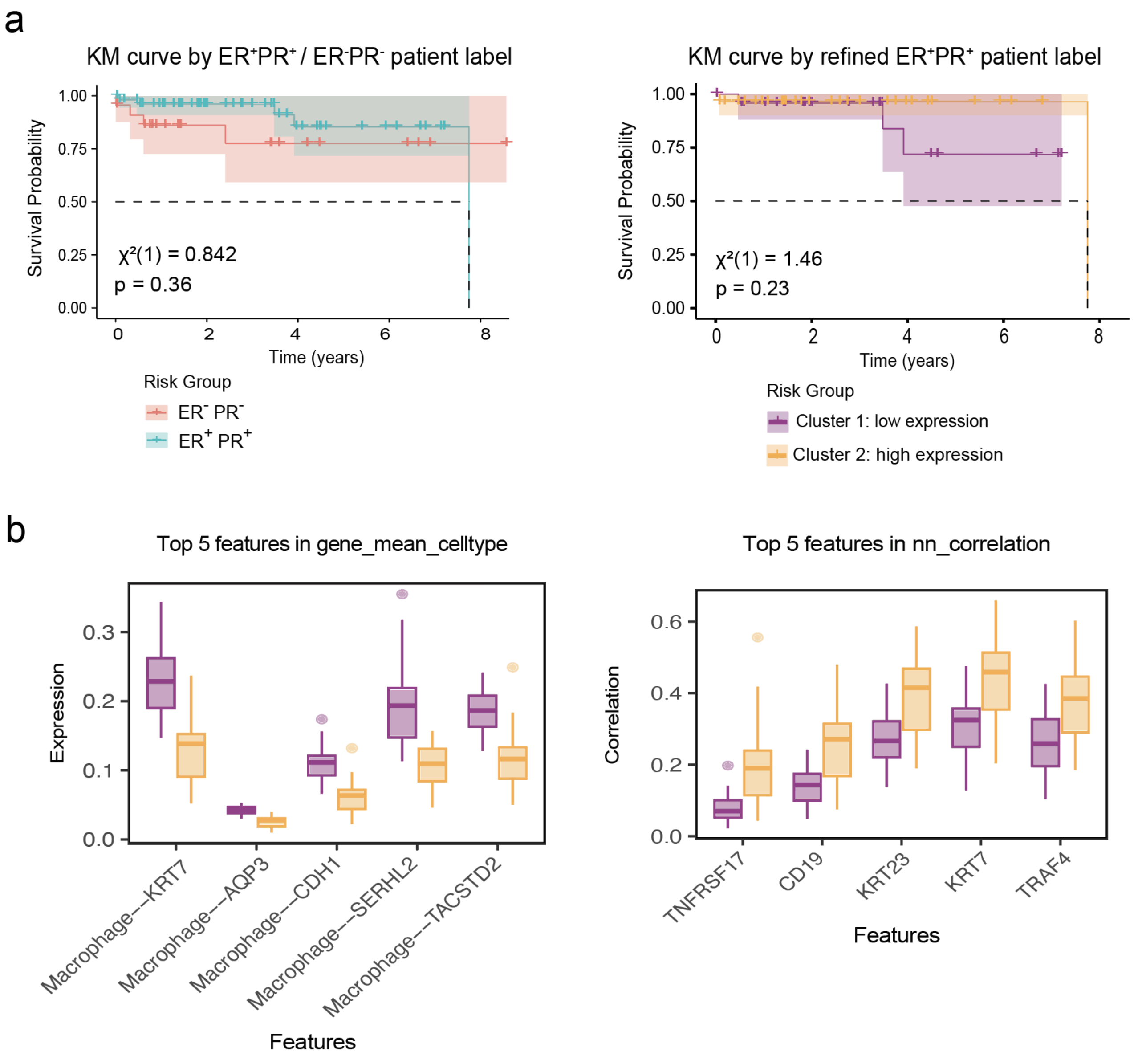

Supplementary Figure S16 KM curves of original patient label and refined patient label. **(a)** The KM curve based on the original ER+/PR+ and ER-/PR- patient group as defined in TCGA [left], and the KM curve based on refined patient clusters within the ER+PR+ patients [right]. Shaded regions represent 95% confidence intervals. A two-sided log-rank test was used to calculate the χ^2 (chi-squared) test statistic and *P* value of the survival difference between the two groups (n = 54). **(b)**, the top 5 features with greatest feature importance in the cluster 1 and cluster 2 (n = 54) classification model based on the cell-type-specific gene mean expression feature type [left], and the top 5 features in the model based on the nearest neighbour correlation feature type [right]. Each boxplot ranges from the first to third quartile with the median as the horizontal line. The lower whisker extends 1.5 times the interquartile range below the first quartile, while the upper whisker extends 1.5 times the interquartile range above the third quartile.

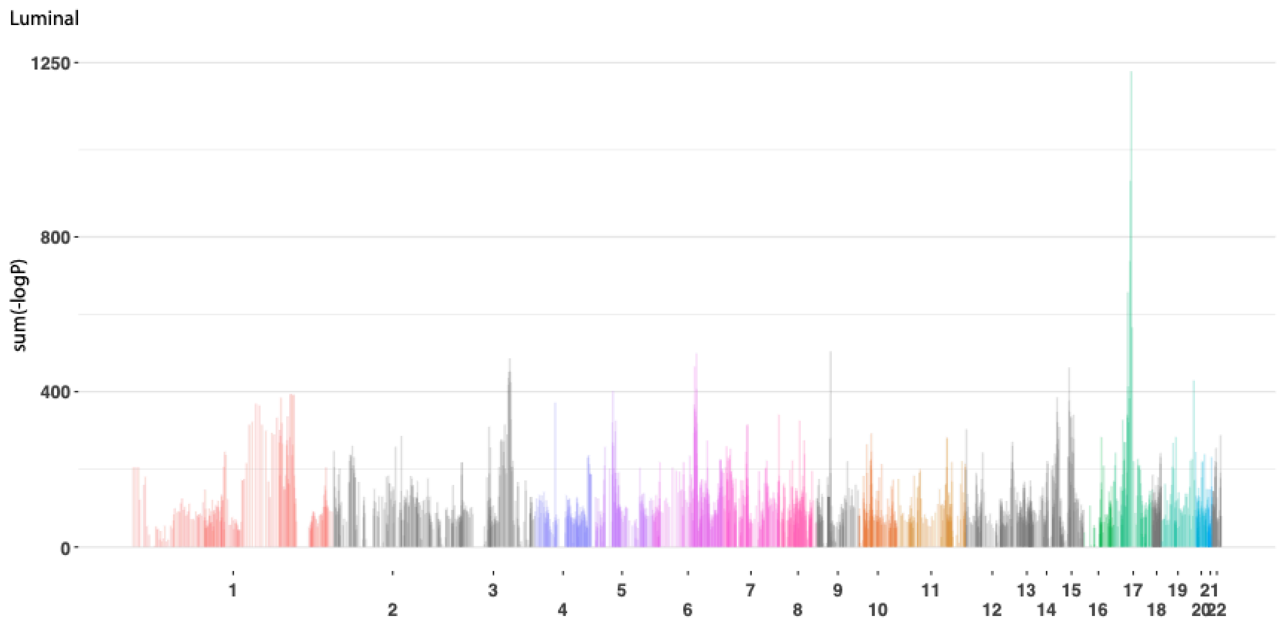

Supplementary Figure S17 Associations of CNAs with spatial gene expression in luminal patients.

Differential spatial gene expression affected by copy number alteration in luminal patients (n = 458) was performed using two-sided t-test. The sum of -log10(P) of each genomic region of the associations between CNAs and spatial gene expression. The *P* value were reported without multiple comparison adjustments.

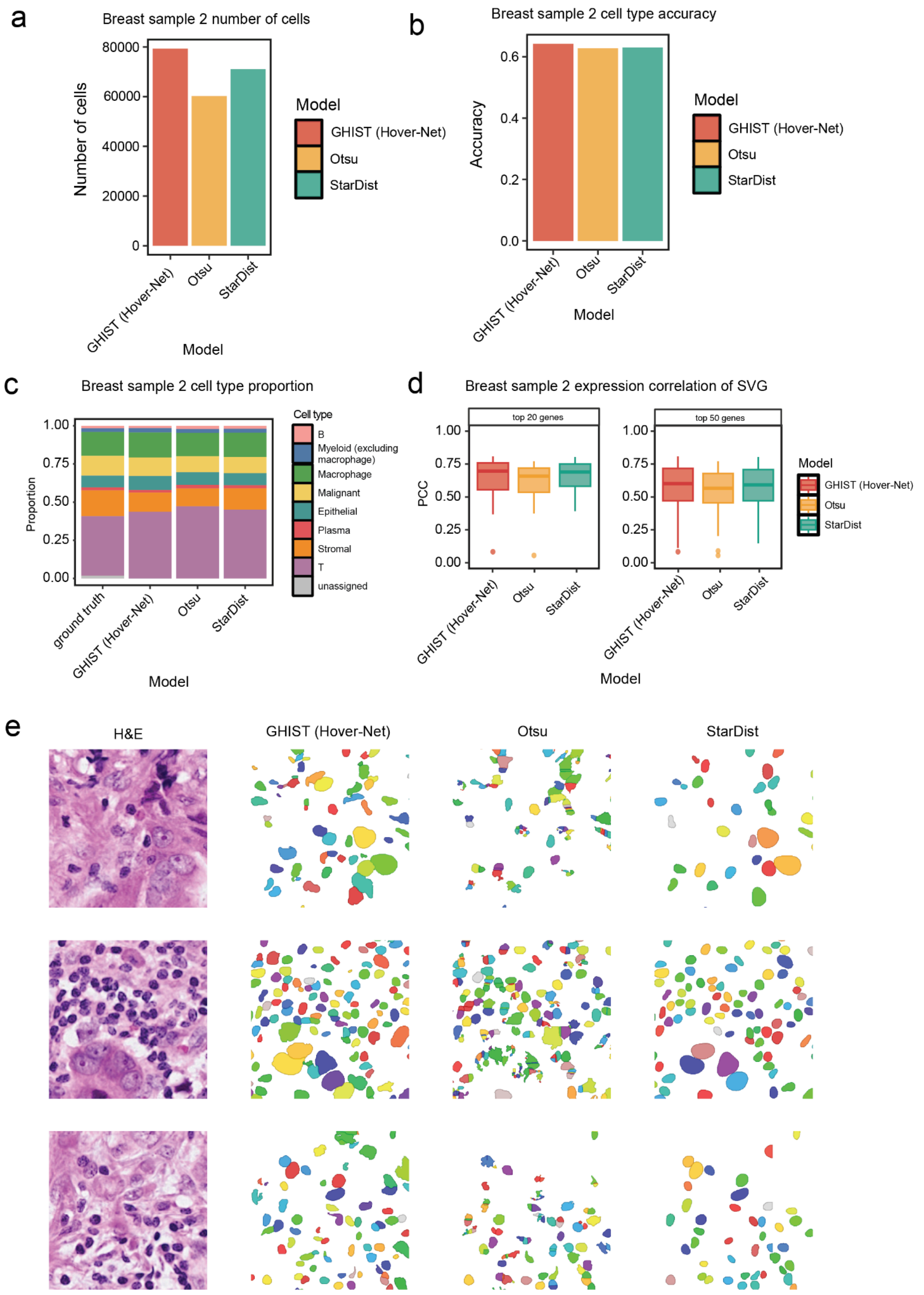
a
Breast sample 2 number of cells
Number of cells
80000
60000
40000
20000
0
GHIST (Hover-Net)
Otsu
StarDist
Model
Model
GHIST (Hover-Net)
Otsu
StarDist
b
Breast sample 2 cell type accuracy
Accuracy
0.6
0.4
0.2
0.0
GHIST (Hover-Net)
Otsu
StarDist
Model
Model
GHIST (Hover-Net)
Otsu
StarDist
c
Breast sample 2 cell type proportion
Proportion
1.00
0.75
0.50
0.25
0.00
ground truth
GHIST (Hover-Net)
Otsu
StarDist
Model
Cell type
B
Myeloid (excluding macrophage)
Macrophage
Malignant
Epithelial
Plasma
Stromal
T
unassigned
d
Breast sample 2 expression correlation of SVG
top 20 genes
top 50 genes
PCC
1.00
0.75
0.50
0.25
0.00
GHIST (Hover-Net)
Otsu
StarDist
Model
Model
GHIST (Hover-Net)
Otsu
StarDist
e
H&E
GHIST (Hover-Net)
Otsu
StarDist

Supplementary Figure S18 Results using different nuclei segmentation methods (Hover-Net, Otsu, and StarDist) for BreastCancer2.
The performance of GHIST was the highest with Hover-Net nuclei segmentations. **(a)** Barplot showing the number of cells predicted by each method. **(b)** Barplot showing the accuracy of cell types of gene expression predicted by GHIST. **(c)** Number of cells in each predicted cell type. **(d)** PCC of the predicted top 20 and top 50 SVGs. Each boxplot ranges from the first to third quartile with the median as the horizontal line. The lower whisker extends 1.5 times the interquartile range below the first quartile, while the upper whisker extends 1.5 times the interquartile range above the third quartile. The sample size corresponds to the number of genes included (either 20 or 50). **(e)** Example nuclei segmentations from the three methods, where Hover-Net appears the most visually accurate, leading to the best performance overall.

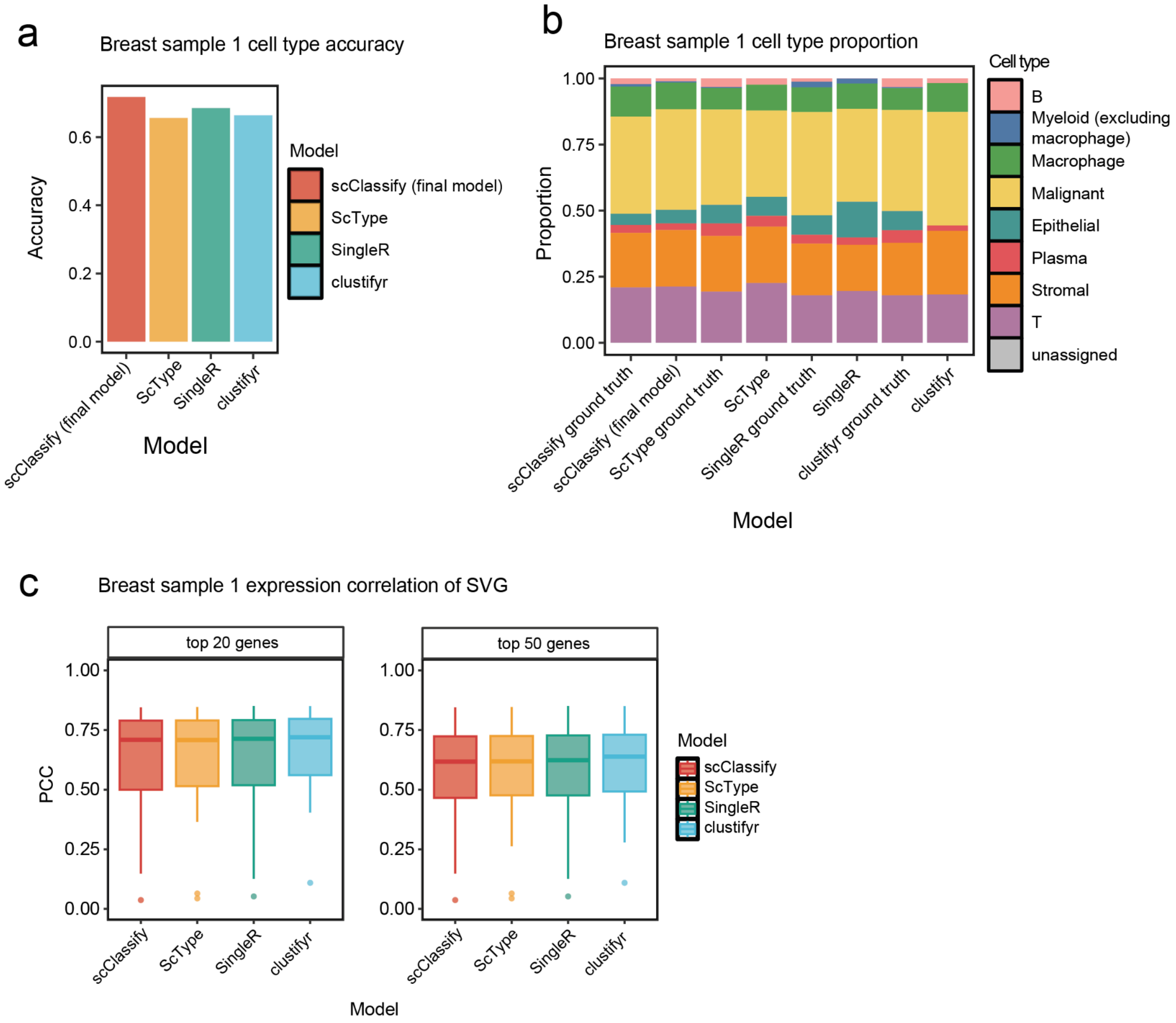

Supplementary Figure S19 Performance of GHIST when trained using cell type labels from different cell type annotation methods on BreastCancer1.
The setting using scClassify had highest cell type accuracy, while the predicted top SVGs were largely consistent across the different methods. **(a)** Accuracy of predicted cell types of the predicted expression from the various settings. **(b)** Predicted cell type proportions. **(c)** PCC of predicted top SVGs. Each boxplot ranges from the first to third quartile with the median as the horizontal line. The lower whisker extends 1.5 times the interquartile range below the first quartile, while the upper whisker extends 1.5 times the interquartile range above the third quartile. The sample size corresponds to the number of genes included (either 20 or 50).

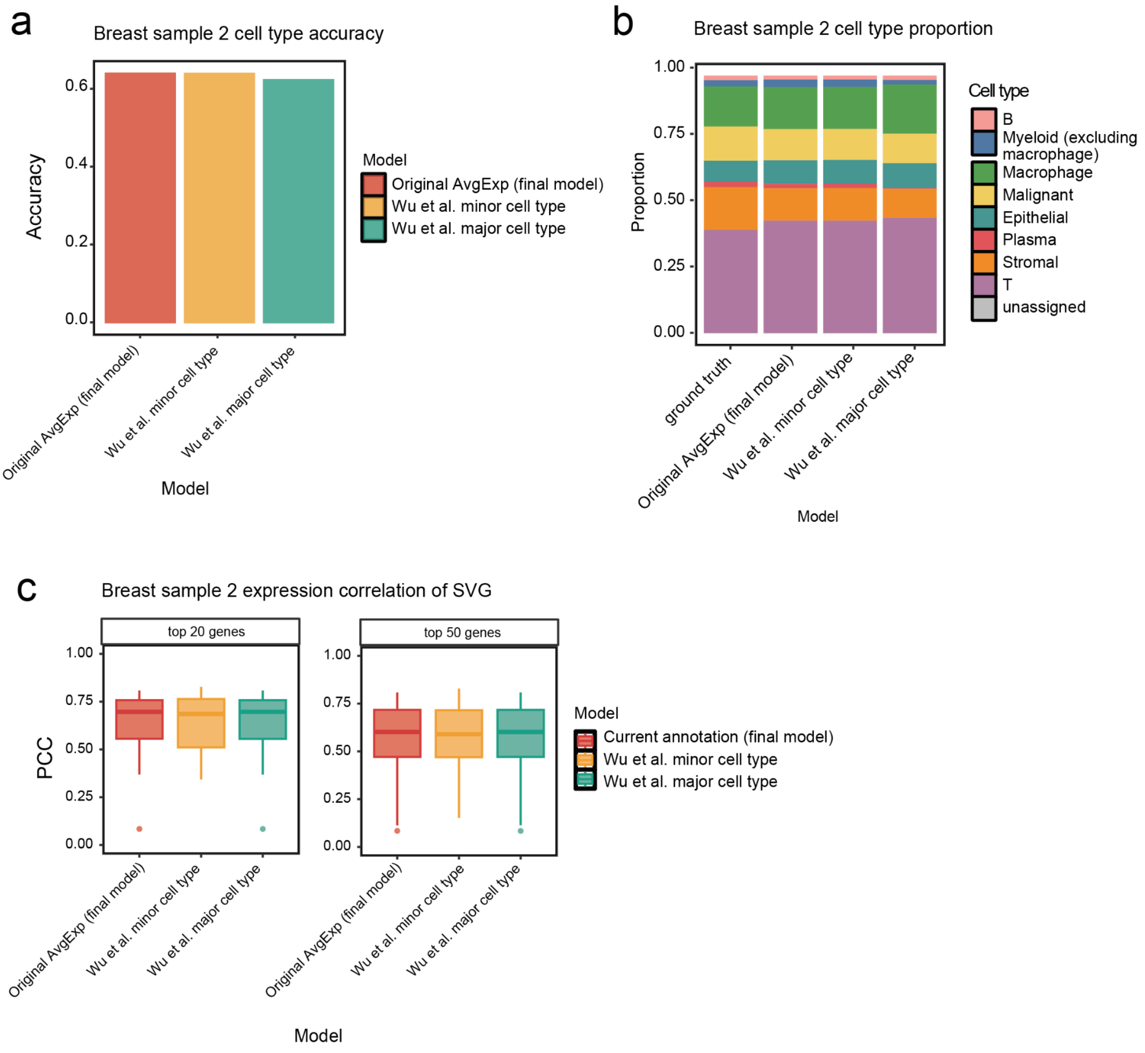

Supplementary Figure S20 Performance of GHIST using averaged cell type expression profiles from different single-cell reference data for BreastCancer2.
Reference Wu et al. major refers to the major cell type category containing 7 cell types, Wu et al. minor refers to a finer cell type category containing 27 cell types. The reference data for the original results contained 15 different cell type profiles. Performance of GHIST was consistent for the different profiles used. **(a)** Accuracy of predicted cell types of the predicted expression from the various settings. **(b)** Predicted cell type proportions. **(c)** PCC of predicted SVGs. Each boxplot ranges from the first to third quartile with the median as the horizontal line. The lower whisker extends 1.5 times the interquartile range below the first quartile, while the upper whisker extends 1.5 times the interquartile range above the third quartile. The sample size corresponds to the number of genes included (either 20 or 50).

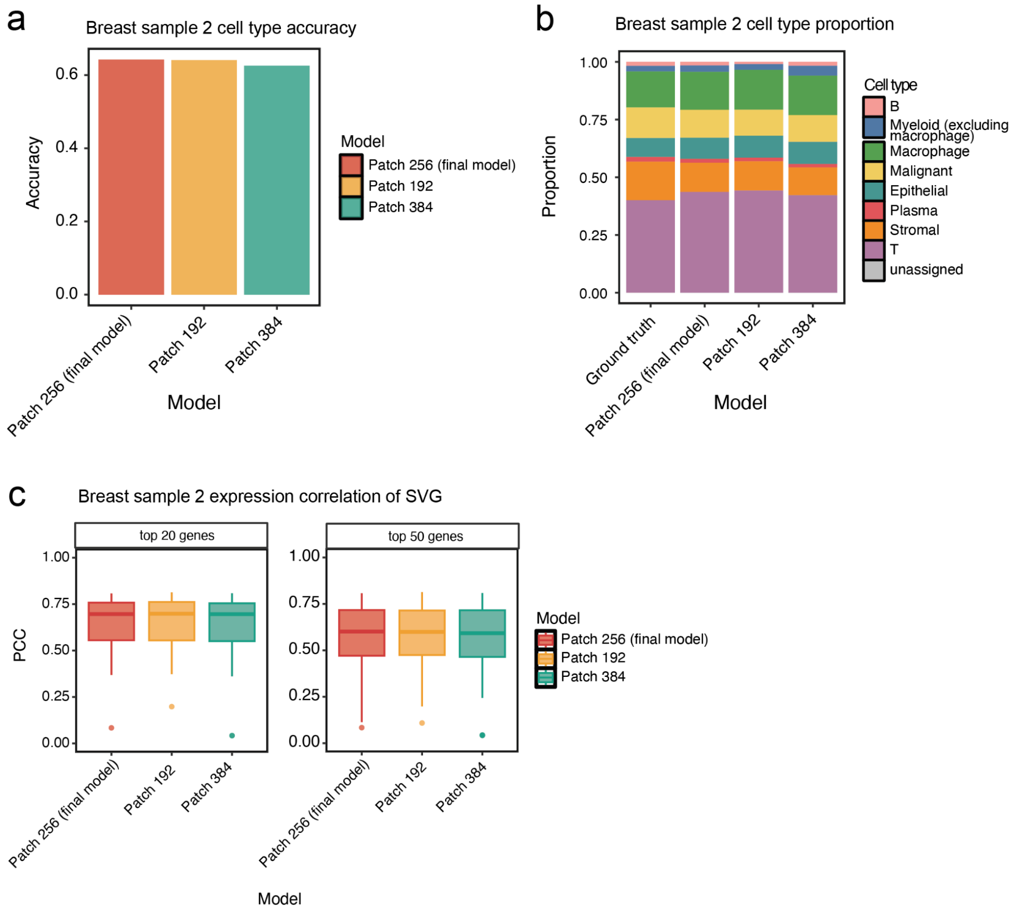

Supplementary Figure S21 Performance for different patch sizes (192×192, 256×256, and 384×384) using BreastCancer2.

(a) Accuracy of predicted cell types of the predicted expression from the various settings. **(b)** Predicted cell type proportions. **(c)** PCC of predicted SVGs. Each boxplot ranges from the first to third quartile with the median as the horizontal line. The lower whisker extends 1.5 times the interquartile range below the first quartile, while the upper whisker extends 1.5 times the interquartile range above the third quartile. The sample size corresponds to the number of genes included (either 20 or 50). The effect of the varying number of cells per patch is minimised during training by normalising the composition of cell types to sum to 1. As the patch size becomes larger, however, predictions become more difficult.

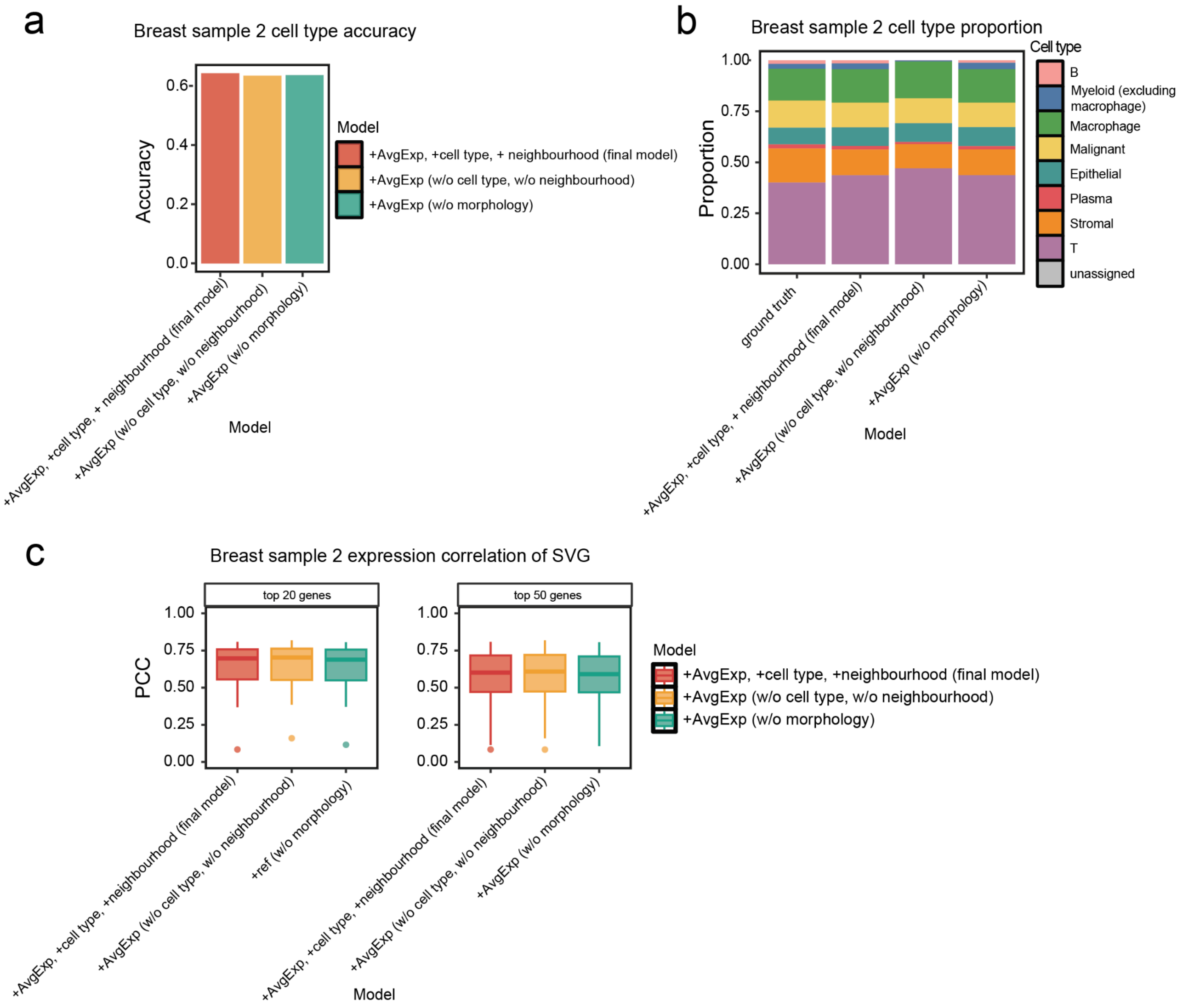

Supplementary Figure S22 Effects of L_{Morph} and $L_{CT,\,class}$.

(a) Accuracy of predicted cell types of the predicted expression from the various settings. **(b)** Predicted cell type proportions. **(c)** PCC of predicted SVGs. Each boxplot ranges from the first to third quartile with the median as the horizontal line. The lower whisker extends 1.5 times the interquartile range below the first quartile, while the upper whisker extends 1.5 times the interquartile range above the third quartile. The sample size corresponds to the number of genes included (either 20 or 50). Performance was improved when both losses were included. Overall predicted cell type accuracy and proportions were worse without $L_{CT,\,class}$ compared to without L_{Morph}, while the correlation of predicted SVGs were worse without L_{Morph} compared to without $L_{CT,\,class}$. Hence, the two losses serve different purposes in GHIST.

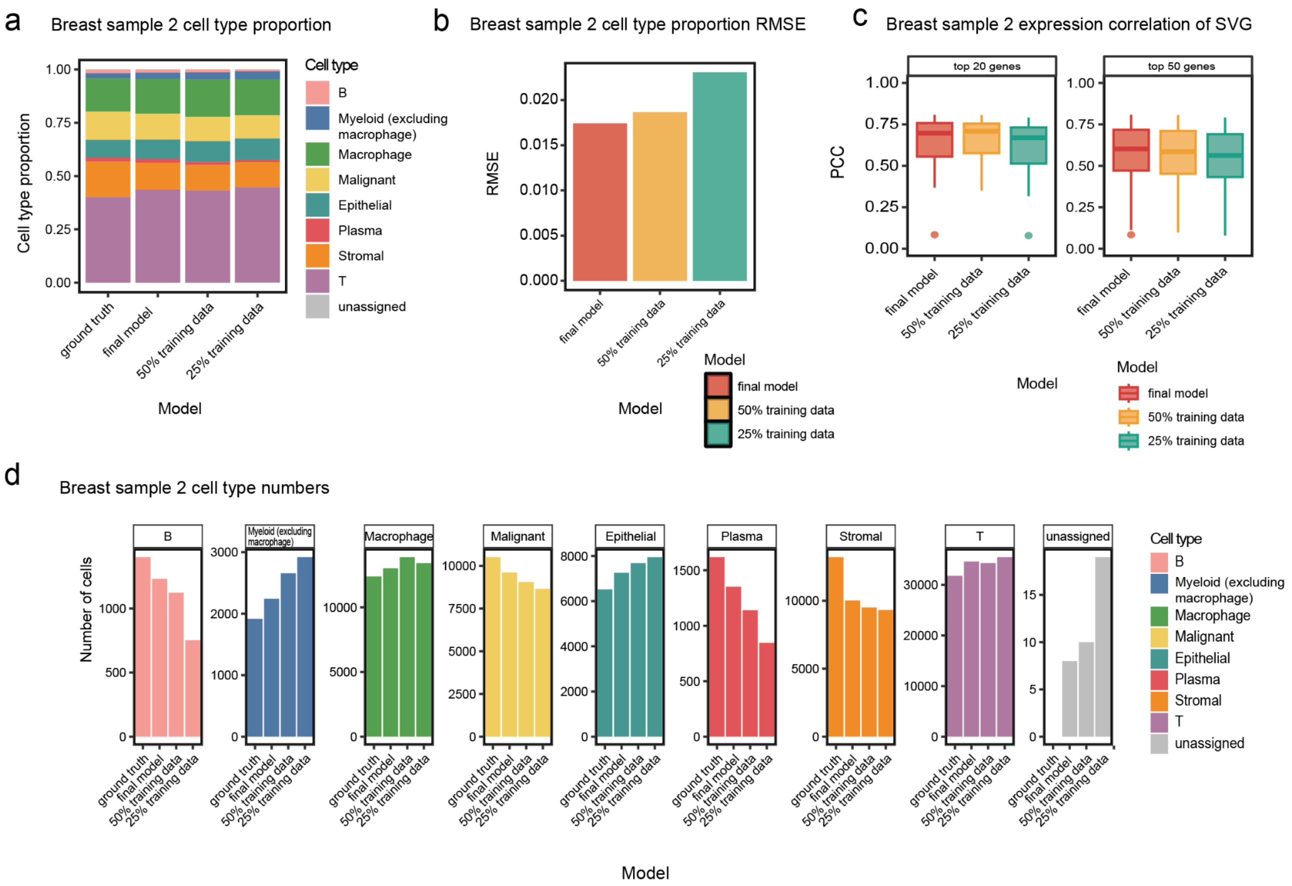

Supplementary Figure S23 Impact of varying amounts of training data.
We observed a reduction in prediction performance with a smaller amount of data used for training the model. The general expectation for deep learning models is that performance will be better with more training data, and vice versa. (a) Predicted cell type proportions. Cell types are predicted from scClassify using predicted single-cell expressions from the various settings mapped spatially across the slide. (b) RMSE of the difference between predicted cell type proportions and ground truth cell type proportions. (c) PCC of predicted SVGs. Each boxplot ranges from the first to third quartile with the median as the horizontal line. The lower whisker extends 1.5 times the interquartile range below the first quartile, while the upper whisker extends 1.5 times the interquartile range above the third quartile. The sample size corresponds to the number of genes included (either 20 or 50). (d) Number of cells for each predicted cell type.

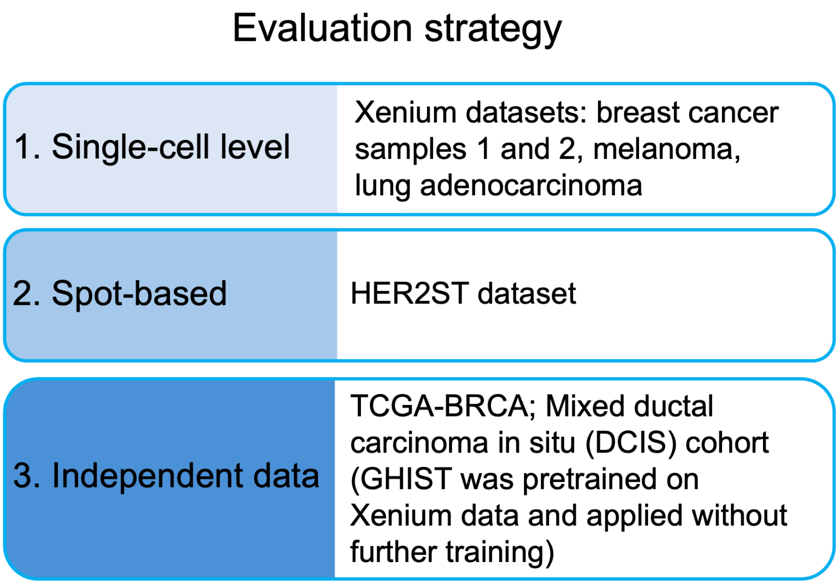

Supplementary Figure S24 Multi-level evaluation strategy.
At single-cell resolution, we utilised a collection of Xenium datasets and examined the predicted gene expression profile against the ground truth gene expression profile. At spot-based resolution, we used the HER2ST dataset and examined the predicted gene expression profile against the ground truth gene expression profile. For independent data, we leveraged the breast cancer cohort from The Cancer Genome Atlas (TCGA) database and an in-house mixed ductal carcinoma in situ (DCIS) cohort. We constructed the single-cell gene expression for the breast cancer cohorts using GHIST (pre-trained), a new data modality that was not previously accessible in the two databases. We evaluated the utility of the predicted single-cell gene expression by constructing patient models.
