# Supplementary material for: Spatial gene expression at single-cell resolution from histology using deep learning with GHIST
Source: Nat Methods. 2025 Sep 15;22(9):1900–10. doi: 10.1038/s41592-025-02795-z (PMC12446070; doi:10.1038/s41592-025-02795-z)
Supplement: Supplementary file 2 — Reporting Summary [file 41592_2025_2795_MOESM2_ESM.pdf]

nature portfolio

Corresponding author(s): Jean Yee Hwa Yang

Last updated by author(s): May 9, 2025

Reporting Summary

Nature Portfolio wishes to improve the reproducibility of the work that we publish. This form provides structure for consistency and transparency in reporting. For further information on Nature Portfolio policies, see our Editorial Policies and the Editorial Policy Checklist.

Statistics

For all statistical analyses, confirm that the following items are present in the figure legend, table legend, main text, or Methods section.

n/a	Confirmed	
☐	☒	The exact sample size (*n*) for each experimental group/condition, given as a discrete number and unit of measurement
☐	☒	A statement on whether measurements were taken from distinct samples or whether the same sample was measured repeatedly
☐	☒	The statistical test(s) used AND whether they are one- or two-sided<br>*Only common tests should be described solely by name; describe more complex techniques in the Methods section.*
☒	☐	A description of all covariates tested
☐	☒	A description of any assumptions or corrections, such as tests of normality and adjustment for multiple comparisons
☐	☒	A full description of the statistical parameters including central tendency (e.g. means) or other basic estimates (e.g. regression coefficient) AND variation (e.g. standard deviation) or associated estimates of uncertainty (e.g. confidence intervals)
☐	☒	For null hypothesis testing, the test statistic (e.g. *F*, *t*, *r*) with confidence intervals, effect sizes, degrees of freedom and *P* value noted<br>*Give P values as exact values whenever suitable.*
☒	☐	For Bayesian analysis, information on the choice of priors and Markov chain Monte Carlo settings
☒	☐	For hierarchical and complex designs, identification of the appropriate level for tests and full reporting of outcomes
☐	☒	Estimates of effect sizes (e.g. Cohen's *d*, Pearson's *r*), indicating how they were calculated

Our web collection on statistics for biologists contains articles on many of the points above.

Software and code

Policy information about availability of computer code

Data collection	TCGA-BRCA RNA-Seq data and histology images were downloaded using the TCGAbiolinks package47 version 2.29.6.<br>TCGA-BRCA clinical (meta data) with variables for defining breast cancer subtypes for samples were downloaded from the TCGAretriever package version 1.9.1.<br>Somatic copy number alteration was downloaded from UCSC Xena (Genomic Data Commons (GDC) TCGA Breast Cancer) (https://xena.ucsc.edu/public).<br>For all other data, they were directly downloaded from the links as listed in the section below. No software was required/used to download the data.
Data analysis	GHIST code is available at https://github.com/SydneyBioX/GHIST, including code for model training, validation, testing, inference<br>BIDCell version 1.0.3 https://github.com/SydneyBioX/BIDCell<br>scClassify version 1.12.0 https://github.com/SydneyBioX/scClassify<br>HoVer-Net version 67e2ce5 https://github.com/vqdang/hover_net<br>torchstain Python package version 1.3.0<br>ST-Net we implemented this in Python version 3.10 and PyTorch version 2.1.1 using hyperparameter values as outlined in the original paper<br>HisToGene version 44ff75b https://github.com/maxpmx/HisToGene<br>GeneCodeR version 6bea8b3 https://github.com/AskExplain/GeneCodeR<br>DeepSpaCE version 5e023ea https://github.com/tmonjo/DeepSpaCE<br>DeepPT we implemented this in Python and PyTorch using hyperparameter values as outlined in the original paper<br>Hist2ST version 7480e5d https://github.com/biomed-AI/Hist2ST<br>iStar version 3cb0e53 https://github.com/daviddaiweizhang/istar<br>Stardist version 0.9.1 https://github.com/stardist/stardist

SKimage (for Otsu) version 0.24.0 https://scikit-image.org/
Giotto rank version 1.1.2
Seurat R package version 5.0.3
ClassifyR R package version 3.9.1
scFeatures version 1.4.0
muscat R package version 1.18.0
ComplexHeatmap R package version 2.20.0
survival R package version 3.6-4
limma R package version 3.60.2
scran R package version 1.32.0
SPARK-X R package version a8b4bf2

For manuscripts utilizing custom algorithms or software that are central to the research but not yet described in published literature, software must be made available to editors and reviewers. We strongly encourage code deposition in a community repository (e.g. GitHub). See the Nature Portfolio guidelines for submitting code & software for further information.

Data

Policy information about availability of data

All manuscripts must include a data availability statement. This statement should provide the following information, where applicable:

- Accession codes, unique identifiers, or web links for publicly available datasets
- A description of any restrictions on data availability
- For clinical datasets or third party data, please ensure that the statement adheres to our policy

All datasets used in this study are publicly available and were downloaded from the following links.
10x Genomics Xenium breast cancer samples 1 and 2, and single-cell data: https://www.10xgenomics.com/products/xenium-in-situ/preview-dataset-human-breast.
10x Genomics Xenium lung adenocarcinoma: https://www.10xgenomics.com/datasets/preview-data-ffpe-human-lung-cancer-with-xenium-multimodal-cell-segmentation-1-standard.
10x Genomics Xenium melanoma: https://www.10xgenomics.com/datasets/human-skin-preview-data-xenium-human-skin-gene-expression-panel-add-on-1-standard.
Melanoma single-cell reference data was downloaded from dbGaP under accession code phs001861.v1.p1 (http://www.ncbi.nlm.nih.gov/projects/gap/cgi-bin/study.cgi?study_id=phs001861.v1.p1).
Lung atlas data from CZI CELLXGENE data portal: https://cellxgene.cziscience.com/collections/6f6d381a-7701-4781-935c-db10d30de293.
HER2ST dataset: https://github.com/almaan/her2st.
NuCLS dataset: https://github.com/PathologyDataScience/BCSS.
TCGA-BRCA: https://portal.gdc.cancer.gov/projects/TCGA-BRCA.
TCGA-BRCA clinical data: https://www.cbioportal.org/study/clinicalData?id=brca_tcga
Somatic copy number alteration for study GDC TCGA Breast Cancer (BRCA) was downloaded from UCSC Xena https://xena.ucsc.edu/.
Default parameter values for Xenium and single-cell reference file: https://github.com/SydneyBioX/BIDCell.

Human research participants

Policy information about studies involving human research participants and Sex and Gender in Research.

Reporting on sex and gender	N/A
Population characteristics	N/A
Recruitment	N/A
Ethics oversight	N/A

Note that full information on the approval of the study protocol must also be provided in the manuscript.

Field-specific reporting

Please select the one below that is the best fit for your research. If you are not sure, read the appropriate sections before making your selection.

☒ Life sciences ☐ Behavioural & social sciences ☐ Ecological, evolutionary & environmental sciences

For a reference copy of the document with all sections, see nature.com/documents/nr-reporting-summary-flat.pdf

Life sciences study design

All studies must disclose on these points even when the disclosure is negative.

Sample size	We validated GHIST on 4 publicly available subcellular spatial transcriptomics datasets. The datasets were captured on different disease types (breast cancer, lung adenocarcinoma, and melanoma), and involved varying numbers of genes (280, 313, 377, 382). The number of cells in each dataset ranged from 47k to 94k. The number of samples was determined by the original authors. All the samples were used.

We also validated GHIST on a publicly available spot-based spatial transcriptomics dataset. The dataset consists of 36 samples of HER2+ breast tissue sections from 8 patients. The number of samples was determined by the original authors. All the samples were used.

Furthermore, we also validated GHIST on TCGA-BRCA data. In our study, we mostly focused on the HER2+ breast cancer subtype from TCGA-BRCA, as this was the subtype of the data (BreastCancer2 and HER2ST) used to train the model and is of clinical interest due to its aggressive nature. We selected the HER2+ patients based on a positive entry in the “lab_proc_her2_neu_immunohistochemistry_receptor_status” metadata column. We supplemented our analysis using luminal breast cancer subtype, using the criteria based based on a previous study (PMID: 39934114): based on either a positive entry in “breast_carcinoma_estrogen_receptor_status” with a positive entry in “breast_carcinoma_progesterone_receptor_status” or a positive entry in “breast_carcinoma_estrogen_receptor_status” with a negative entry in “breast_carcinoma_progesterone_receptor_status”. Overall we considered 92 TCGA HER2+ samples and 461 TCGA luminal patients consisting of matched images, RNA-seq data, somatic copy number data, and clinical data.

We also validated GHIST on DCIS and invasive breast cancer tumour microarray (TMA) data from 44 female patients who were diagnosed with invasive breast cancer. Their use in this project was reviewed and approved by the Western Sydney Local Health District Human Research Ethics Committee (Approval number 2019/ETH02688). The number of samples was determined by the original study. All the samples were used.

Data exclusions	Four TCGA-BRCA patients did not have CNA data, and was therefore excluded from the analysis between somatic CNA and nearest neighbour correlation.
Replication	External validation is used to demonstrate replicability (using a model trained on Xenium data then applied to TCGA data). Cross-validation was used where possible to evaluate the performance and application of GHIST, including for survival analysis. Furthermore, we validated GHIST on 4 publicly available subcellular spatial transcriptomics datasets (across 3 diseases), a publicly available spot-based spatial transcriptomics dataset, and TCGA data.
Randomization	N/A. We didn't have multiple experimental groups across biological samples.
Blinding	N/A. We did not have multiple experimental groups or treat the samples in a way to induce a measurable effect.

Reporting for specific materials, systems and methods

We require information from authors about some types of materials, experimental systems and methods used in many studies. Here, indicate whether each material, system or method listed is relevant to your study. If you are not sure if a list item applies to your research, read the appropriate section before selecting a response.

Materials & experimental systems

n/a	Involved in the study
☒	☐ Antibodies
☒	☐ Eukaryotic cell lines
☒	☐ Palaeontology and archaeology
☒	☐ Animals and other organisms
☒	☐ Clinical data
☒	☐ Dual use research of concern

Methods

n/a	Involved in the study
☒	☐ ChIP-seq
☒	☐ Flow cytometry
☒	☐ MRI-based neuroimaging
